# Butenolides and triterpenoids from the fungus *Pallidohirschioporus biformis* (syn. *Trichaptum biforme*): isolation, structure determination and bioactivity profile

**DOI:** 10.3389/ffunb.2026.1737900

**Published:** 2026-06-03

**Authors:** Suratno Suratno, Csenge Anna Felegyi-Tóth, Elżbieta Studzińska-Sroka, Viktor Papp, Imre Boldizsár, Annamária Kincses, Tamás Gáti, Przemysław Zalewski, Szabolcs Béni, Attila Ványolós

**Affiliations:** 1 Department of Pharmacognosy, Semmelweis University, Budapest, Hungary; 2 Center for Pharmacology and Drug Research & Development, Semmelweis University, Budapest, Hungary; 3 Department of Pharmacognosy and Biomaterials, Poznan University of Medical Sciences, Poznan, Poland; 4 Department of Botany, Hungarian University of Agriculture and Life Sciences, Budapest, Hungary; 5 Department of Plant Anatomy, Institute of Biology, Eötvös Loránd University, Budapest, Hungary; 6 Institute of Pharmacognosy, University of Szeged, Szeged, Hungary; 7 Servier Research Institute of Medicinal Chemistry (SRIMC), Budapest, Hungary; 8 Integrative Health and Environmental Analysis Research Laboratory, Department of Analytical Chemistry, Institute of Chemistry, Eötvös Loránd University, Budapest, Hungary

**Keywords:** antioxidant, butenolides, butyrylcholinesterase, hyaluronidase, *Hymenochaetales*, steroids, triterpenes, tyrosinase

## Abstract

*Pallidohirschioporus biformis* is a common annual polypore distributed in subtropical to temperate regions of the Northern Hemisphere. Extensive mycochemical analysis of the methanol extract of *P. biformis* resulted in the isolation of eight secondary metabolites (1-8). The structure determination of the fungal metabolites was carried out by one- and two-dimensional NMR and MS analysis. Among them biformolide (1) is a new natural product, while compound 2 (9-(2-methoxy-3, 4-dimethyl-5-oxofuran-2-yl)nonanoic acid) is reported from natural sources for the first time. Besides compounds 1-2, two other butenolides, namely hydroxydihydrobovolide (3) and schumannione (4), 4-hydroxy-trans-2-nonenoic acid (5), betulin (6), and two ergostane-type derivative, ergosterol peroxide (7) and ergosterol peroxide glucoside (8) have been identified. The isolates as well as the chloroform, n-hexane, and methanol extracts of *P. biformis* were evaluated for their potential bioactivities in several *in vitro* assays, including hyaluronidase, acetyl- and butyrylcholinesterase (AChE and BuChE), alpha-glucosidase, antibacterial, tyrosinase inhibitory and antioxidant tests. The bioassay results for the three fungal extracts suggest that hyaluronidase could be a potential enzymatic target of *P. biformis* extracts. Of the secondary metabolites tested, compound 8 showed the highest hyaluronidase inhibitory activity (91%), whereas compound 6 exhibited moderate inhibition (32%), and the remaining compounds demonstrated little to no activity. The anticholinesterase assays revealed a notable difference between the inhibitory effects of *P. biformis* on AChE and BuChE. Although the chloroform and methanol extracts exhibited significant BuChE inhibition, they showed no inhibitory activity against AChE. Our findings identify hydroxydihydrobovolide (3) as the primary bioactive compound responsible for the observed inhibition of butyrylcholinesterase activity. This study is the first comprehensive report on the chemical composition of wild-growing *P. biformis* sporocarps, providing an in-depth investigation into the isolation and structural characterization of the species’ most distinctive biologically active compounds.

## 1 Introduction

While numerous macrofungi have been extensively studied for their bioactive compounds and therapeutic applications, many species remain poorly explored, and their pharmacological potential is still largely unknown. As Wu et al. (2019) emphasize, among the documented macrofungal taxa in China, 692 species are recognized as medicinal, yet many have not been thoroughly examined for their pharmacological properties, representing a vast and largely untapped reservoir for drug discovery.

The *Hymenochaetales* is one of the largest orders of *Basidiomycota* and comprises a diverse assemblage of fungi, most of which are wood-inhabiting (He et al., 2024). The order is particularly notable because it includes several medicinally important species, such as *Inonotus obliquus*, *Phellinus igniarius*, and *Sanghuangporus baumii* (Ern et al., 2024; Luan et al., 2022; Zhou et al., 2020). In recent years, a growing number of studies have focused on previously underexplored hymenochaetoid polypores, including *Fuscoporia torulosa*, *Hirschioporus fuscoviolaceus, Inonotus nidus-pici*, *Phylloporia boldo*, *Porodaedalea chrysoloma*. These studies have aimed to identify novel bioactive compounds by characterizing chemical profiles and detecting key secondary metabolites with potential biological activities (Béni et al., 2021; Garádi et al., 2021; Riquelme et al., 2020; Sárközy et al., 2020; Suratno et al., 2025).

In contrast to members of the *Hymenochaetaceae* (Ghobad-Nejhad et al., 2024), the genus *Trichaptum* sensu lato remains mycochemically underexplored within the *Hymenochaetales*. It currently comprises 55 species distributed across four families and seven genera (Zhou et al., 2023, 2026). Among these, *Pallidohirschioporus biformis* (syn. *Trichaptum biforme*) is one of the best-studied species from both biotechnological and medicinal perspectives. This widespread annual polypore occurs in subtropical to temperate regions of the Northern Hemisphere and grows on various angiosperm hosts (Zhou et al., 2023). As a white-rot fungus, *P*. *biformis* plays an important role in cellulose degradation and has considerable biotechnological potential. Recently, Xie et al. (2025) sequenced its genome using a combination of approaches. Their work provided a more comprehensive view of its functional complexity and clarified the genetic basis of its lignocellulose-degrading capacity.

Regarding its chemical composition, Robbins et al. (1947) first reported biformin, a polyacetylene compound isolated from cultures of *P. biformis* and exhibiting antibacterial activity. Other studies revealed that this species was an excellent source of sesquiterpenes of different types. Cadinane sesquiterpenes, trichapargins A and B, were isolated from the cultures of *P. biformis*. Trichapargin A showed weak activity to SW480 (Tang et al., 2017). Yang et al. (2014) identified cadinane-type sesquiterpenes and 13-carbon *γ*-lactones from the cultures of this species. They were evaluated for their cytotoxicity against human cancer cell lines, but none of them exerted significant activity. In another study, Yang et al. (2013) described two drimane sesquiterpenoids in the cultures of *P. biformis.* with no significant cytotoxicity against human cancer cell lines.

While the cultures of *P. biformis* have been extensively studied, we know little about the chemical profile of the fruiting bodies of the wild growing *P. biformis*. A recent study by Habibi et al. (2024) reported the presence of ergosterol, ergosterol peroxide and 9, 11-dehydroergosterol peroxide from the sporocarps of this species.

The promising health-related properties of *P. biformis* have been investigated in previous studies (Robbins et al., 1947; Shnyreva et al., 2018; Yang et al., 2017). This fungus exhibits promising medicinal properties, particularly immunomodulatory and potential anticancer activity, primarily attributed to its polysaccharides (Habibi et al., 2024).

## 2 Material and methods

### 2.1 General experimental procedures

The flash chromatography (FC) separations were performed using a CombiFlash^®^ NextGen 300+ (Teledyne Isco, Lincoln, NE, USA) instrument equipped with UV and UV-Vis detectors. The 30 g RediSep^®^ Rf Gold High Performance C18 (Teledyne Isco, Lincoln NE, USA) and a 5.5 g RediSep^®^ Rf Gold C18Aq (Teledyne Isco, Lincoln, Nebraska, USA) columns were employed as reversed-phase chromatography columns. The 80 g RediSep^®^ Rf Gold High Performance Silica (Teledyne Isco, Lincoln, NE, USA) column was utilized as a normal phase chromatography column.

The reverse-phase HPLC separations were carried out using a Waters^®^ Alliance 2695 HPLC Separations Module equipped with Waters 996 Photodiode Array Detector (Waters Corporation, Milford, MA, USA) and Kinetex^®^ EVO C18, 100 Å, 5 μm, 150 × 10 mm i.d. (Phenomenex Inc, Torrance, CA, USA) column as the stationary phase. Fraction collections in the HPLC separations were performed manually. The chemicals used were purchased from Sigma-Aldrich Kft. (Budapest, Hungary) and Molar Chemicals (Halásztelek, Hungary).

The high-resolution mass spectrometry measurements were carried out by using Dionex Ultimate 3000 UHPLC system (3000RS diode array detector, TCC-3000RS column thermostat, HPG-3400RS pump, SRD-3400 solvent rack degasser, and WPS-3000TRS autosampler) (Thermo Fischer Scientific, Waltham, MA, USA) linked to an Orbitrap Q Exactive Focus Mass Spectrometer with an electrospray ionization source (Thermo Fischer Scientific, Waltham, MA, USA). The ionization source was operated in both positive and negative ionization modes, with automatic optimization of operational parameters using built-in software. The following experimental parameters were used: spray voltage (+) 3500 V, spray voltage (−) 2500 V; capillary temperature 320 °C; sheath gas (N2) 47.5 °C; auxiliary gas (N2) 11.25 units; spare gas (N2) 2.25 arbitrary units. The full scan resolution was set to 70,000, and the scan range was between 10 and 1500 *m/z* units. Parent ion fragmentation was performed with normalized collision energies of 15%, 30%, and 45%.

^1^H (500.1 MHz) and ^13^C (125.6 MHz) NMR spectra of compounds were recorded at room temperature on a Bruker 500 Avance NEO NMR spectrometer equipped with cryogenic probe head. Amounts of approximately 0.5–5 mg of compounds were dissolved in 0.6 ml of methanol-*d_4_* and transferred into a 5 mm NMR sample tube. The NMR solvent (methanol- *d_4_*; 99.8% D) was purchased from Eurisotop (Saint-Aubin, France). Chemical shifts are given on the *δ*-scale and are referenced to the solvent (methanol-*d_4_*: *δ*_C_ = 49.1 and *δ*_H_ = 3.31 ppm). Pulse programs of all experiments (one-dimensional ^1^H, ^13^C, DEPTQ, DEPT-135, sel-TOCSY, two-dimensional ^1^H,^1^H-COSY, gs-HSQC, edited gs-HSQC, gs-HMBC and band-selective HSQC and band-selective HMBC) were taken from the Bruker software library.

### 2.2 Mushroom material

The fruiting bodies of *P. biformis* were gathered in the Mecsek Mountains, in the vicinity of Kisújbánya (Hungary) on June 6^th^, 2021. The collected fungal samples were cleaned from any pollution including soil contaminants and plant parts, then stored on −20 °C. A voucher specimen (No. PB-202107) has been deposited at the Department of Pharmacognosy, Semmelweis University, Hungary.

### 2.3 Extraction and isolation

The air-dried fruiting bodies of *P. biformis* (624 g) were ground, soaked in 5 L methanol for one hour and subsequently percolated at room temperature using 8 L methanol and 14.5 L of reused methanol obtained upon the evaporation of the extract. The methanolic fungal extract was concentrated in vacuo, dissolved in 50% (v/v) methanol, then subjected to solvent-solvent partition using *n*-hexane (6 × 200 mL) and chloroform (5 × 200 mL), and ethyl acetate (5 × 200 mL), consecutively. The combined *n*-hexane and chloroform phases were dried leading to 12.13 g and 4.72 g dry extracts, respectively, then further subjected to chromatographic separation.

The *n*-hexane fraction (12.13 g) was initially divided into two parts, then each part was separated using normal-phase flash chromatography (NP-FC) applying a *n*-hexane-acetone eluent system (0-100% v/v acetone gradient elution). The fractions obtained from the NP-FC separations with similar characteristics were combined based on TLC monitoring. The first part of the *n*-hexane fraction separation afforded 23 combined fractions (PH1/1-23), while the second part afforded 26 combined fractions (PH2/1-26). In the coming step fractions PH1/13, PH1/14, PH1/15 and PH1/16 were combined (673.1 mg), and subsequently purified by a reversed-phase flash chromatography (RP-FC) method applying a water-methanol as mobile phase with 10-100% v/v methanol gradient elution, which led to 14 combined fractions (PH1/13-16/1 – PH1/13-16/14). The targeted fraction PH1/13-16/10 (129.9 mg) was initially separated using a reversed-phase high-performance liquid chromatography (RP-HPLC) method with a gradient elution of water-acetonitrile (acetonitrile 81-84% v/v), then finally purified by RP-HPLC method using an isocratic method of water-acetonitrile 17:83 v/v, leading to the isolation of compound 7 (5.2 mg). The fractions PH2/24 and PH2/25 were combined (180.3 mg), then purified by RP-FC using a water-methanol solvent system (10 to 100% v/v of methanol gradient elution), yielding compound 8 (5.2 mg).

Regarding the chloroform fraction (4.72 g) it was initially separated by NP-FC using a gradient elution of *n*-hexane-acetone by increasing acetone 0-100% v/v, affording 16 combined fractions (PC1 - PC16). By applying an RP-FC method, fraction PC5 (77.7 mg) was further separated using a water-methanol eluent system as mobile phase (0-100% v/v methanol gradient elution), resulting 27 combined fractions (PC5-1 – PC5-27). Fraction PC5-14 (7.0 mg), PC5-15 (15.8 mg) and PC5-22 (9.0 mg) were further purified by RP-HPLC using water-acetonitrile system (31-33% v/v, 35-38% v/v, and 80-90% v/v acetonitrile gradient elution, respectively), giving compounds 1 (4.1 mg), 3 (9.3 mg) and 6 (1.1 mg), respectively. The final purification of fraction PC7 (159.1 mg) was performed by RP-FC using a solvent system of water-methanol (0 to 100% v/v methanol gradient elution), leading to 21 combined fractions (PC7-1 – PC7-21). Distinctly, PC7-9 (7.8 mg), PC7-11 (8.5 mg) and PC7-13 (4.9 mg) were subjected to RP-HLPC purification applying a mobile phase of water-acetonitrile (18-38% organic solvent content), yielding compounds 4 (1.8 mg), 5 (3.6 mg) and 2 (1.1 mg), respectively.

Biformolide (1): colorless oil; HR-ESI-MS (+) *m/z* 229.1427 [M + H]^+^ (229.1440 calcd for C_12_H_21_O_4_ Δ 3.1 ppm); HR-ESI-MS (–) *m/z m/z* 227.1286 [M–H]^–^ (227.1283 calcd for C_12_H_19_O_4_ Δ 3.5 ppm); ^1^H and ^13^C NMR data, see **Table 1**; HR-ESI-MSMS (+) (CID = 15%, 30%, 45%) *m/z* 211.1322, 183.1375, 151.1111, HRESI-MSMS (–) (CID = 15%, 30%, 45%) *m/z* 209.1178, 195.1014, 183.1382, 167.1064, 151.1115. The absolute configuration of compound 1 could not be determined due to the limited quantity of the isolated material, which precluded the application of chiroptical (e.g. VCD) or derivatization-based (e.g. Mosher) methods.

**Table 1:** NMR assignments for compounds **1** and **2.**.

Atom number	Compound 1			Compound 2		
	*δ* ^13^C (ppm)	*δ* ^1^H (ppm, multiplicity)	HMBC correlations	*δ* ^13^C (ppm)	*δ* ^1^H (ppm, multiplicity)	*HMBC correlations*
1	174.5	–	–	173.7	–	
2	126.8	–	–	128.5	–	
3	159.5	–	–	158.3	–	
4	109.4	–	–	111.9	–	
5	84.0	3.41 m 1H	3, 4, 7, 10	36.3	1.72, 1.95 m, 2H	4,
6	30.6	1.38, 1.61 m, 2H	7, 8	23.5	1.16,1.26 m, 2H	8
7	29.6	1.36, 1.51 m, 2H	6, 8, 9	29.5-30.5	1.24-1.37 m, 8H	5, 7, 8, 9,10
8	23.8	1.35 m, 2H	6, 9			
9	14.4	0.93 t, *J* = 7.2 Hz, 3H	7, 8			
10	60.7	3.43 s, 3H	5			
11	11.8	1.99 br. s, 3H	(1), 2, 3, 4, 12	26.3	1.59 m, 2H	9
12	8.3	1.79 q, *J* = 1.1 Hz, 3H	1, 2, 3, (4), 11	35.9	2.25 br. s, 2H	10
13	–	–	–	n.d.	–	
14	–	–	–	50.2	3.07 s, 3H	4
15	–	–	–	10.6	1.90 q, *J* = 1.1 Hz, 3H	(1), 2, 3, 4, 16
16	–	–	–	8.0	1.83 q, *J* = 1.1 Hz, 3H	1, 2, 3, (4), 15

9-(2-Methoxy-3,4-dimethyl-5-oxofuran-2-yl)nonanoic acid (2): colorless oil; HR-ESI-MS (+) *m/z* 299.1843 [M + H]^+^ (299.1859 calcd for C_16_H_27_O_5_ Δ 3.1 ppm); HR-ESI-MS (–) *m/z m/z* 297.1708 [M–H]^–^ (297.1702 calcd for C_16_H_25_O_5_ Δ 3.9 ppm); ^1^H and ^13^C NMR data, see **Table 1**; HR-ESI-MSMS (+) (CID = 15%, 30%, 45%) *m/z* 267.1581, 249.1478, 231.1373, 221.1528, 213.1266, 203.1425, 185.1319, 175.1476, 137.0594, HRESI-MSMS (–) (CID = 15%, 30%, 45%) *m/z* 283.1548, 265.1445, 253.1808, 239.1648, 221.1542.

### 2.4 Biological activity assays

#### 2.4.1 Acetyl- and butyrylcholinesterase inhibitory activity

The acetyl- and butyrylcholinesterase (AChE and BuChE) inhibition activity were evaluated according with the method described Ellman et al. (1961) with some modification (Studzińska-Sroka et al., 2022). The inhibition of BuChE was evaluated for the extracts and all isolated compounds, while the inhibition of AChE was only evaluated for the extracts, not for the isolated compounds. The extracts and compounds were dissolved in DMSO (20 mg/mL and 40 mg/mL for extracts; 20 mg/mL for compounds). Briefly, 5.0 µL of the sample, 60.0 µL of TRIS-HCl buffer (50 mM, pH = 8), and 30 µL of AChE (0.2 U/mL) or BuChE (0.2 U/mL) were mixed. Subsequently, the plate was incubated for 5 min. at 25 °C with shaking (500 rpm). Next, 30.0 µL of acetylthiocholine iodide (ATChI) 1.5 mM or butyrylthiocholine iodide (BTChI) 1.5 mM and 125.0 µL of 5,5′-dithiobis(2-nitrobenzoic acid) (0.3 mM with 10 mM NaCl and 2 mM MgCl_2_·6H_2_O) were added and incubated with shaking (500 rpm) for 30 min (AChE) or 20 min (BuChE) at the same temperature condition (25 °C). The blanks of samples were prepared with 30 µL of the buffer instead of AChE or BuChE. The control sample contained DMSO instead of the tested substance. The blank of control contained 5 µL of DMSO instead of samples and 30 µL of the buffer instead of AChE or BuChE solution. Absorbance was measured at 405 nm (Multiskan GO 1510, Thermo Fisher Scientific, Vantaa, Finland). The final result was calculated as the average of tree results for extracts (n = 3), and two results for compounds (n=2). The results were expressed as % of BuChE inhibition activity ± SD.


$$\text{Inhibition of AChE}/\text{BuChE}\left[\%\right]=100-\frac{\left(\text{As }-\text{Abs}\right)}{\left(\text{Ac}-\text{Abc}\right)}\times 100$$

where: As is the absorbance of the sample, Abs is the absorbance of the blank of the sample, Ac is the absorbance of the control and Abc is the blank of the control.

Galantamine hydrobromide at concentrations ranging from 0.0625 to 0.5 mg/mL (0.218 – 1.74 mM) was used as a standard for the butyrylcholinesterase (BuChE) inhibition assay, and its activity was expressed as the IC_50_ value calculated from the plotted graph of BuChE inhibition (%) versus standard concentration. The results are presented as the mean of four independent measurements (n = 4).

The chemicals used in the acetyl- and butyrylcholinesterase inhibitory activity assays were purchased from Sigma–Aldrich (St. Louis, MO, USA).

#### 2.4.2 Hyaluronidase inhibition activity

The hyaluronidase inhibition activity was performed according to the previously described method (Studzińska-Sroka et al., 2024b). Briefly, 25.0 µL of incubation buffer (acetate buffer pH 4.5, with 77 mM NaCl and 1 mg/mL albumin), 25.0 µL of enzyme solution (30 U/mL), 10.0 µL of the tested extract (5–20 mg/mL) or compounds (10 mg/mL), and 15.0 µL of acetate buffer were mixed in the well. After 15 min of incubation (37 °C) with shaking at 500 rpm, 25.0 µL of hyaluronic acid (HA) solution (0.3 mg/mL) was added and incubated for 45 min with shaking (37 °C). After this time, 200.0 µL of cetyltrimethylammonium bromide (CTAB) solution (2.5%) in 2% sodium hydroxide was added. After 10 min of incubation without shaking (at room temperature), the absorbance (λ = 600 nm) was measured using a plate reader (Multiskan GO 1510, Thermo Fisher Scientific, Vantaa, Finland). The final result was calculated as the average of two results for extract (n = 2) and three results for compounds (n = 3). The results were expressed as % of hyaluronidase activity inhibition ± SD.


$$\text{Inhibition of hyaluronidase}\left[\%\right]=\frac{\left(\text{TS}-{\text{TE}}_{\text{blank}}\right)}{\left({\text{TH}}_{\text{blank}}-{\text{TE}}_{\text{blank}}\right)}\times 100$$

where: TS is the absorbance of sample; TE_blank_ is the absorbance of the enzyme + examined substance without HA; TH_blank_ is the absorbance of the HA + examined substance without the enzyme.

Escin at concentrations ranging from 6–9 mg/mL (5.30 – 7.95 mM) was used as a standard for the hyaluronidase inhibition assay, and its activity was expressed as the IC_50_ value calculated from the plotted graph of hyaluronidase inhibition (%) versus standard concentration. The results are presented as the mean of four measurements (n = 4).

#### 2.4.3 Tyrosinase inhibition activity

The tyrosinase inhibition activity was evaluated using the spectrophotometric method (Lim et al., 2009) with some modifications described previously (Studzińska-Sroka et al., 2021). The extracts and compounds were dissolved in DMSO to obtain a concentration of 10 mg/mL (extracts), and 5 mg/mL (compounds). Briefly, 25 µL of the sample, 75 µl of 0.1 M phosphate buffer (pH 6.8), and 50 µL of tyrosinase solution (192 U/mL in phosphate buffer) were mixed. Next, the samples were incubated at room temperature (25 °C) for 10 min with shaking (500 rpm). Subsequently, 50 µL of L-DOPA (2 mM in phosphate buffer) was added and incubated for 20 min with shaking (500 rpm) at the same temperature condition (25 °C). The blanks of samples were prepared using 50 µL of the buffer instead of L-DOPA solution. The control contained DMSO instead of the tested substances. The control blank contained 25 µL of DMSO instead of samples and 50 µL of the buffer instead of L-DOPA solution. Absorbance was measured at 475 nm (Multiskan GO 1510, Thermo Fisher Scientific, Vantaa, Finland). The final result was calculated as the average of three measurements (n = 3). The results were expressed as % of tyrosinase inhibition activity ± SD.


$$\text{Inhibition of tyrosinase}\left[\%\right]=100-\frac{\left(\text{As}\;-\text{Abs}\right)}{\left(\text{Ac}-\text{Abc}\right)}\times 100$$

where: As is the absorbance of the sample, Abs is the absorbance of the blank of the sample, Ac is the absorbance of the control and Abc is the blank of the control.

Azelaic acid at concentrations ranging from 3.2 - 12.8 mg/mL (17.0 – 68.0 mM) was used as a standard for the tyrosinase inhibition assay, and its activity was expressed as the IC_50_ value calculated from the plotted graph of tyrosinase inhibition (%) versus standard concentration. The results are presented as the mean of four measurements (n = 4).

#### 2.4.4 α-glucosidase inhibition activity

The extracts were dissolved in DMSO to obtain a concentration of 10 mg/mL. The spectrophotometric, modified method was used (Studzińska-Sroka et al., 2024a). Briefly, 25 µL of the sample, 50 µL of 0.1 M phosphate buffer (pH 6.8), and 50 µL of α-glucosidase solution (0.5 U/mL in phosphate buffer) were mixed. Next, the samples were incubated (37 °C) for 15 min with shaking (500 rpm). Subsequently, 25 µL of 4-nitrophenyl-D-glucopyranose solution (pNPG, 5 mM in phosphate buffer) was added and incubated for 20 min with shaking (500 rpm) at the same temperature condition (37 °C). After incubation 100 µL of sodium carbonate (IV) (0.2 M in distilled water) was added. The blanks of samples were prepared using 50 µL of the buffer instead of enzyme solution. The control sample contained DMSO instead of the tested substances. The control blank contained 25 µL of DMSO instead of samples and 50 µL of the buffer instead of enzyme solution. Absorbance was measured at 405 nm (Multiskan GO 1510, Thermo Fisher Scientific, Vantaa, Finland). The final result was calculated as the average of two measurements (n = 2). The results were expressed as % of α-glucosidase inhibition activity ± SD.


$$\text{Inhibition of }\alpha \text{-glucosidase}\left[\%\right]=100-\frac{\left(\text{As}-\text{Abs}\right)}{\left(\text{Ac}-\text{Abc}\right)}\times 100$$

where: As is the absorbance of the sample, Abs is the absorbance of the blank of the sample, Ac is the absorbance of the control and Abc is the blank of the control.

Acarbose at concentrations ranging from 1.25–20 mg/mL (1.94 – 30.98 mM) was used as a standard for the α-glucosidase inhibition assay, and its activity was expressed as the IC_50_ value calculated from the plotted graph of α-glucosidase inhibition (%) versus standard concentration. The results are presented as the mean of four measurements (n = 4).

#### 2.4.5 Antioxidant activity (ABTS assay)

A previously described ABTS analysis was used to determine the antiradical activity with slight modifications (Chanaj-Kaczmarek et al., 2015). Briefly, 10.0 μl of fractions samples (5–20 mg/mL, dissolved in DMSO) were mixed with 200.0 μl of ABTS^•+^ solution (7 mM ABTS^•+^ was prepared in 2.45 mM potassium persulfate solution and diluted with water to absorbance value ~0.77). The mixture was shaken in the dark at 500 rpm for 30 minutes at room temperature. Absorbance was measured at 734 nm using a plate reader (Multiskan GO 1510, Thermo Fisher Scientific, Vantaa, Finland). The control contained 10.0 μl of DMSO and 200.0 μl of ABTS^•+^ solution. The blank was the mixture of 10 μl of DMSO and 200 μl distillate water. The final result was calculated as the average of three measurements (n = 3). The results were expressed as % of a radical cation scavenging activity ± SD.


$$\%\text{ Antioxidant activity}=100-\frac{\left(\text{As}-\text{Ab}\right)}{\left(\text{Ac}\;-\text{Ab}\right)}\times 100$$

where: As is the absorbance of the sample, Ac is the absorbance of the control and Ab is the blank. Vitamin C at concentrations ranging from 0.025 - 0.200 µg/mL (1.42 to 11.36 µM) was used as a standard, and its activity was expressed as the IC_50_ value calculated from the plotted graph of ABTS scavenging activity (%) versus standard concentration. The results are presented as the mean of four measurements (n = 4).

#### 2.4.6 Antibacterial activity

*Staphylococcus aureus* MRSA ATCC 43300, *Enterococcus faecalis* ATCC 29212, *Bacillus subtilis* ATCC 6633, *Escherichia coli* ATCC 35218, *Klebisella pneumoniae* ATCC 700603, *Pseudomonas aeruginosa* ATCC 27853, and *Moraxella catarrhalis* ATCC 25238 were purchased from Microbiologics (St. Cloud, MN, USA) and used for the antibacterial assays.

The effects of increasing concentrations of the extracts on bacterial growth (the determination of the minimal inhibitory concentration, MIC) were tested in 96-well flat-bottomed microtiter plates (Weinstein et al., 2020). The compounds were diluted in 100 μL of Mueller-Hinton medium. 10–^4^ dilution of an overnight bacterial culture in 100 μL of medium were then added to each well, except for the medium control wells. The plates were further incubated at 37 °C for 18 h; at the end of the incubation period, the results were visually inspected to determine the MIC values of the compounds. DMSO was used as a negative control to rule out any potential antibacterial effects of the solvent.

## 3 Results and discussion

**Figure 1:**
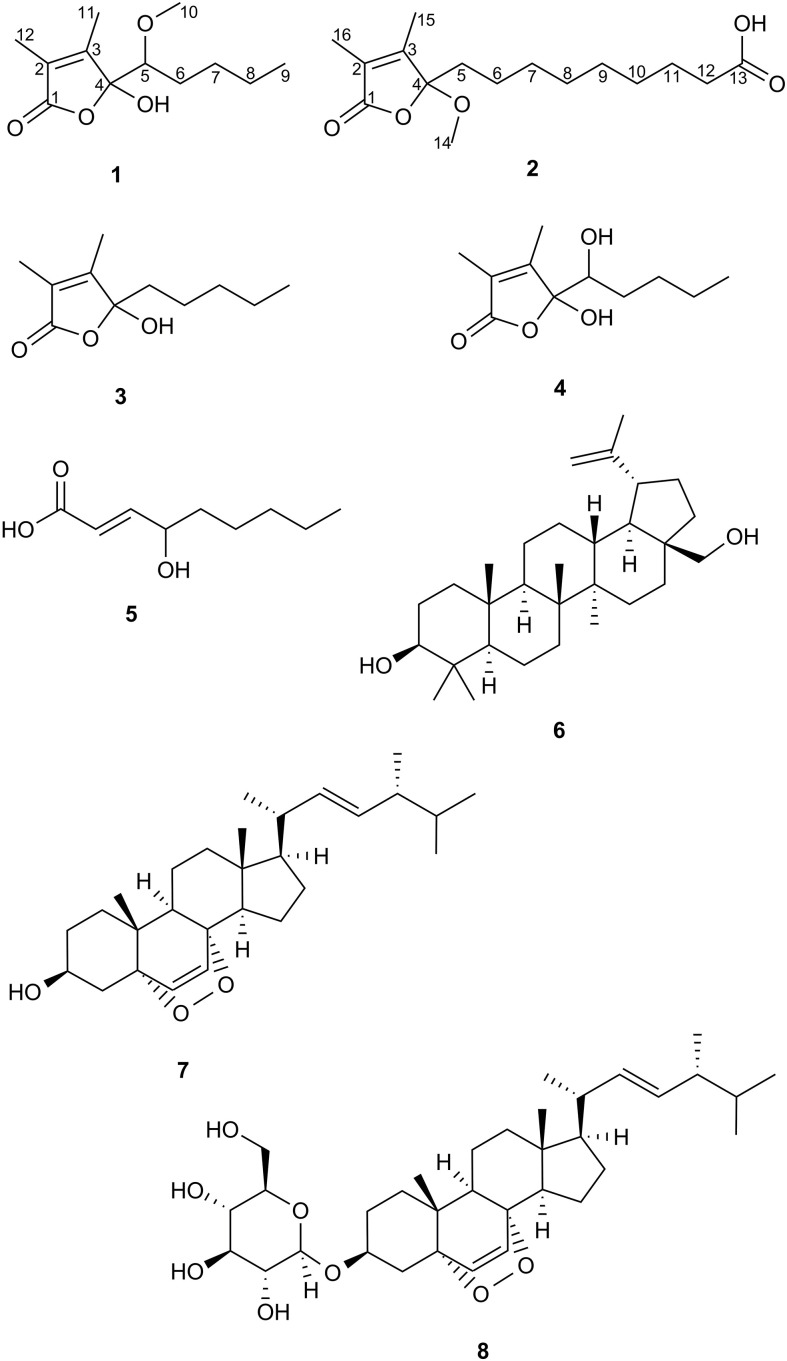
Structures of compounds isolated from *P. biformis*.

Detailed chemical analysis of the methanol extract obtained from the air-dried fruiting bodies of *P. biformis* led to the identification of eight secondary metabolites (1-8) (**Figure 1**). The ground mushroom material was extracted by percolation at room temperature using methanol. The methanol extract was concentrated under vacuum, the dried extract was redissolved in 50% (v/v) methanol then was first subjected to solvent-solvent partition between aqueous MeOH and *n*-hexane, followed by extraction with chloroform. The resulted *n*-hexane and chloroform extracts were purified using normal and reversed phase flash column chromatography. Final purification of fungal metabolites was carried out by reversed-phase HPLC.

**Figure 2:**
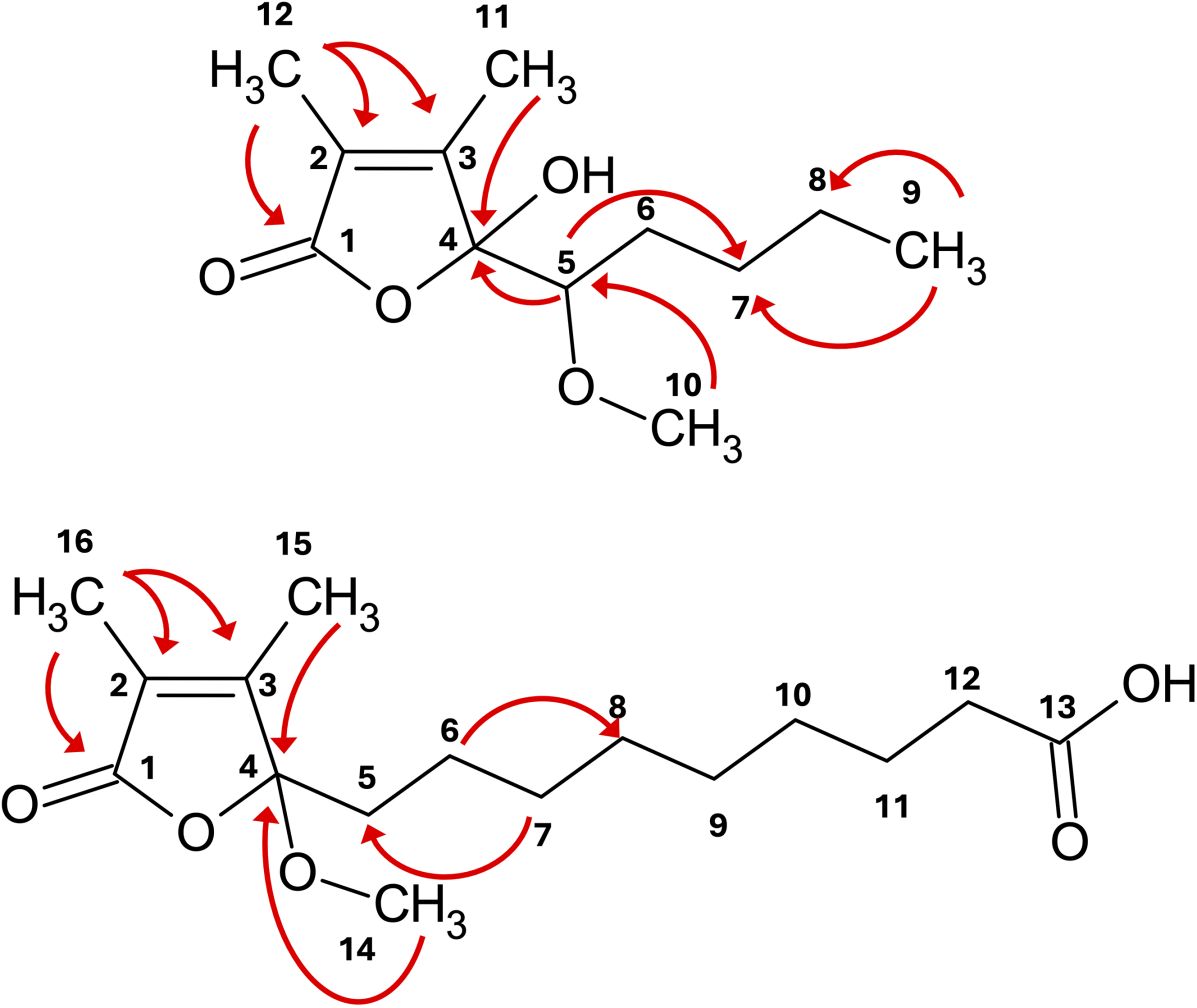
Atom numbering and key HMBC correlations of compounds 1 (top) and 2 (bottom).

Compound 1 was assigned to the molecular formula of C_12_H_20_O_4_ based on HRMS analysis. The ^1^H NMR spectrum in methanol-*d*_4_ revealed four methyl groups: a triplet at 0.93 ppm corresponding to a terminal aliphatic methyl, a broad singlet at 3.43 ppm indicative of a methoxy group, and two additional methyls at 1.79 and 1.99 ppm. The relatively low ^13^C chemical shifts of these latter methyls (8.3 and 11.8 ppm) suggest attachment to sp²-hybridized carbons. Analysis of the ^13^C NMR and DEPT-edited HSQC spectra identified one methine and three methylene carbons at 84.0, 30.6, 29.6, and 23.9 ppm, respectively, indicating an oxymethine (OCH at 3.41 ppm) and three aliphatic CH_2_ groups. Key HMBC correlations (**Figure 2**) allowed the elucidation of the molecular connectivity. The two- and three-bond correlations of the olefinic methyls confirmed their vicinal positions and the presence of a neighboring carbonyl and acetal carbon, consistent with a dimethylated butenolide core. The aliphatic side chain attached to the acetal carbon was established through HMBC correlations between the oxymethine at position 5 and the acetal carbon of the lactone, as well as correlations to the methoxy group and the saturated butyl chain. These data together defined the (planar) structure of the new compound 1, named biformolide. However, although the NMR data established the connectivity, the stereochemical features of the molecule could not be fully resolved. Due to the limited amount, neither the absolute nor the relative configuration could be determined. Consequently, the stereochemistry of compound 1 remains unresolved and will be addressed in future studies when sufficient material becomes available.

The structure elucidation of compound 2 was relatively straightforward, as it shared several structural motifs with compound 1. The presence of two olefinic methyl groups at 1.83 and 1.90 ppm, together with an adjacent carbonyl carbon at 173.7 ppm and an acetal carbon at 111.9 ppm, indicated the same dimethylated butenolide core, which was confirmed by the corresponding HMBC correlations (**Figure 2**). A three-bond HMBC correlation between the methoxy protons and the acetal carbon further supported the direct attachment of these groups. The primary differences between compounds 1 and 2 were observed in the aliphatic side chain. Compound 2 exhibited four distinct and well-resolved methylene groups, two of which contained diastereotopic protons with ^13^C chemical shifts at 36.3 and 23.5 ppm, and two - bearing chemically equivalent protons - at ^13^C chemical shifts of 26.3 and 35.9 ppm. In addition, four overlapping CH_2_ signals were present as strongly superimposed resonances. HMBC correlations allowed the assignment of the diastereotopic methylenes to positions 5 and 6, which were connected to the overlapping CH_2_ units. The side chain was further extended by two well-resolved methylenes with equivalent protons at 1.59 ppm (position 11) and 2.25 ppm (position 12). The combined NMR and MS data confirmed that the aliphatic chain was terminated by a carboxylic acid function, however due to extreme line broadening that carbon could not be detected by NMR. In conclusion, the structure elucidation led to the identification of compound 2 as 9-(2-methoxy-3,4-dimethyl-5-oxofuran-2-yl)nonanoic acid known as a product from chemical synthesis (Kawazoe et al., 2023) but it is isolated for the first time from natural sources. The complete NMR resonance assignment of 1 and 2 can be found in **Table 1**.

Compounds 3–8 were structurally characterized based on NMR spectroscopic and MS data and confirmed by comparing them to those of reported earlier: hydroxydihydrobovolide (3) (Xu et al., 2017), schumannione (4) (Rob et al., 2020), 4-hydroxy-trans-2-nonenoic acid (5) (Lainer et al., 2020), betulin (6) (Liu et al., 2020), ergosterol peroxide (7) (Garádi et al., 2021) and ergosterol peroxide glucoside (8) (Takaishi et al., 1991).

Compounds 1–4 belong to the group of γ-butenolides which are versatile small lactones containing a γ-lactone ring with a C=C bond, found in several natural sources including plants, fungi, and bacteria. Butenolides play important roles as signaling molecules (e.g., karrikins) (Kamran et al., 2024), flavor/aroma compounds (e.g., sotolon) (Tokitomo et al., 1980), and hold biomedical promise (e.g. paraconic acids displaying both antitumor and antibiotic activities) (Bandichhor et al., 2005). Their structure–activity diversity makes them valuable scaffolds in both basic research and applied sciences.

Compound **3** known as hydroxydihydrobovolide was isolated for the first time from burley tobacco (Fujimori et al., 1978), then identified in other species including the evergreen and epiphytic vine *Raphidophora decursiva* (Zhang et al., 2001) and the tropical marine sponge of *Callyspongia samarensis* (Resuello et al., 2018). In previous studies, hydroxydihydrobovolide (3) inhibited HIV-1 replication in a green fluorescent protein (GFP)-based reporter cell line (HOG.R5) (Zhang et al., 2005), however did not show significant antimalarial activity (Zhang et al., 2001).

Schumannione (4) is known from the leaves of *Schumannianthus dichotomus* and investigated for its phytotoxic activity using a monocot plant, timothy (*Phleum pretense*) and a dicot cress (*Lepidium sativum*) as test plant species. It demonstrated significant growth inhibitory activity against timothy and cress seedlings at concentration of 10 mM (Rob et al., 2020).

Compound **5** is 4-hydroxy-trans-2-nonenoic acid reported as a lipid oxidation degradation product identified among others in poppy seed extracts (Lainer et al., 2020). It has been found that 4-hydroxy-trans-2-nonenoic acid is a major product of the detoxification process of 4-hydroxy-trans-2-nonenal which is a neurotoxic product of lipid peroxidation whose levels are elevated in multiple neurodegenerative diseases and CNS trauma (Murphy et al., 2003). Betulin (**6**) is a multifunctional pentacyclic triterpene with potent anticancer, anti-inflammatory, metabolic, antiviral, and tissue-healing properties, documented in several *in vitro*, *in vivo*, and in some clinical trials (Adepoju et al., 2023; Ebeling et al., 2014; Król et al., 2015; Pavlova et al., 2003). Isolates 7–8 are common ergostane derivatives with a widespread distribution in the fungal kingdom displaying a variety of biological activities including anticancer, anti-inflammatory, antiviral and immunomodulatory properties (Duan et al., 2021; He et al., 2018; Ling et al., 2024; Zhabinskii et al., 2022).

**Figure 3:**
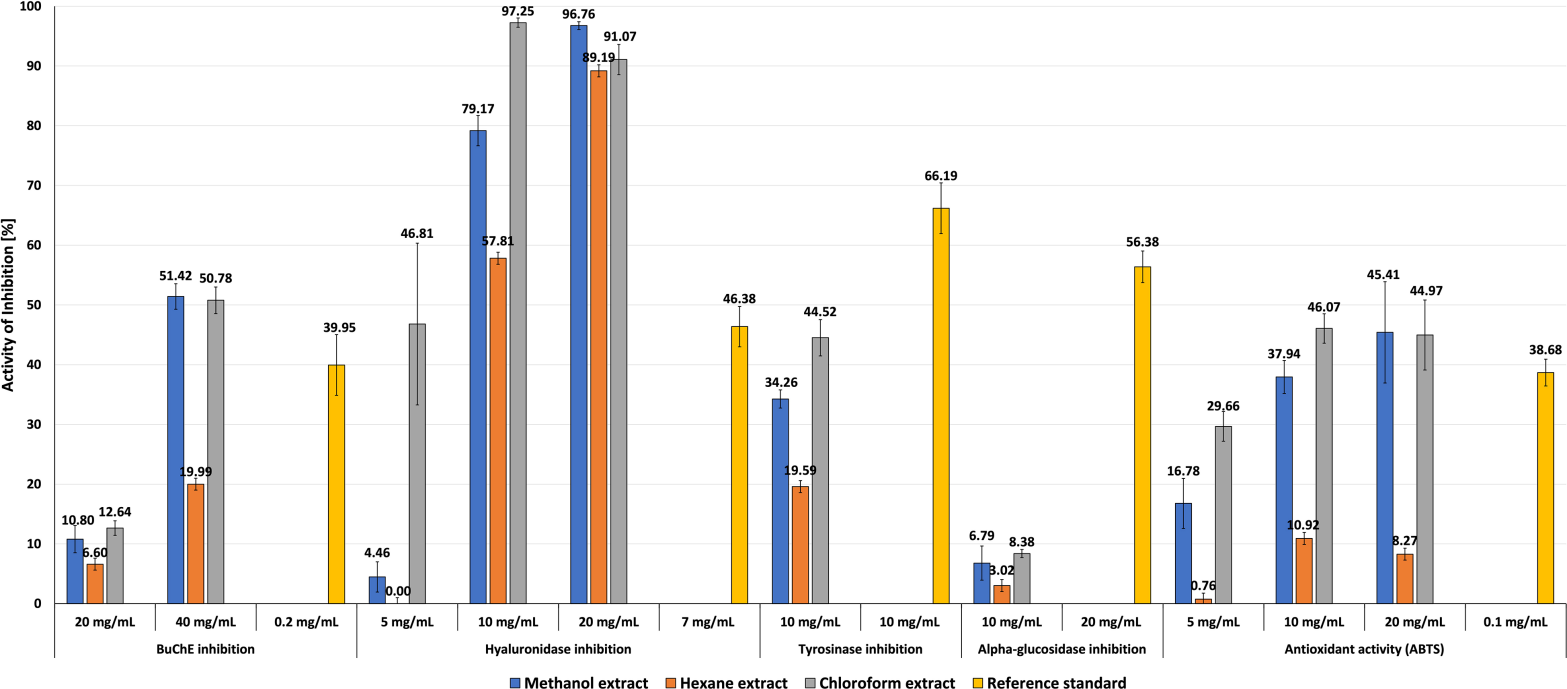
Biological activity of the extracts of *P. biformis*. *Reference standard compounds: galantamine for BuChE inhibition; escin for hyaluronidase inhibition; azelaic acid for tyrosinase inhibition; acarbose for alpha-glucosidase inhibition; and vitamin C for antioxidant activity (ABTS).

**Figure 4:**
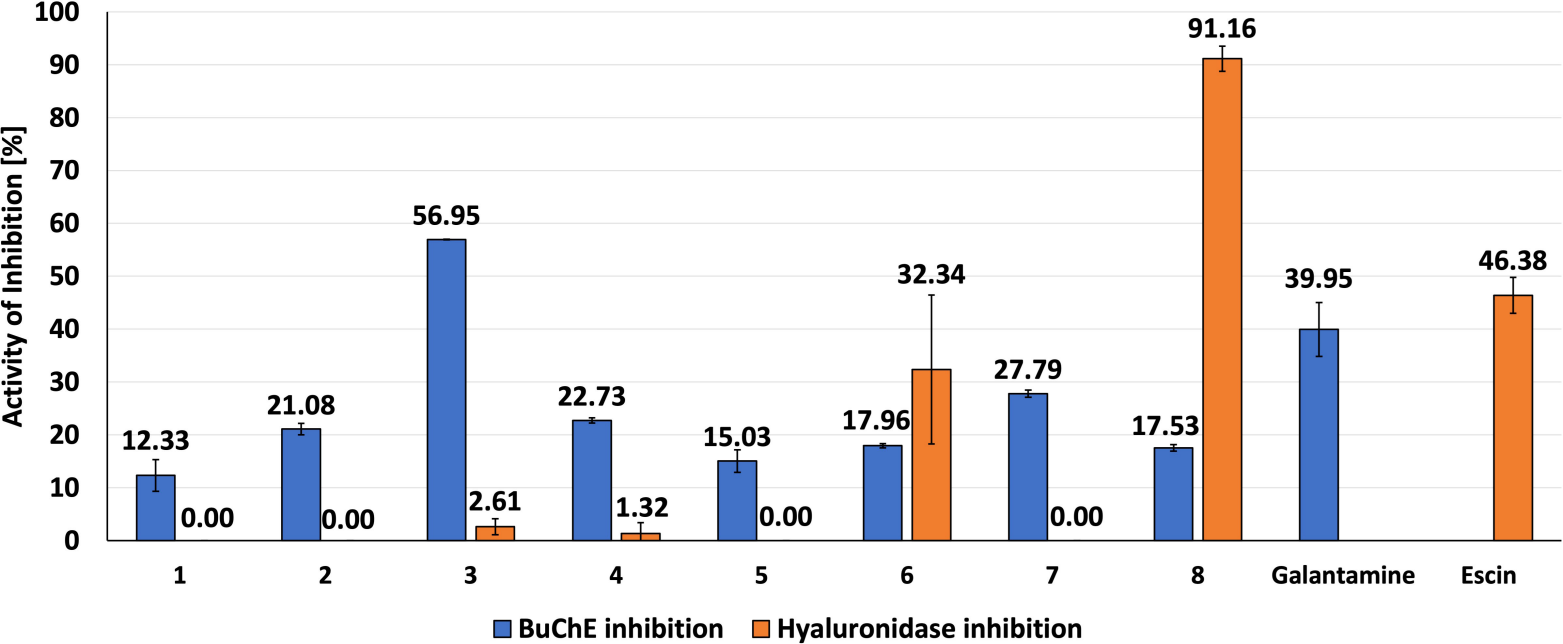
BuChE and hyaluronidase inhibitory activity of compounds 1-8. *Compound concentration: 20 mg/mL for BuChE inhibition; and 10 mg/mL for hyaluronidase inhibition. **Reference standard concentration: 0.2 mg/mL of galantamine; and 7 mg/mL escin.

To explore the potential biological activities of *P. biformis* several assays have been performed on the methanol, *n*-hexane and chloroform extracts of this species, including enzyme activity assays on acetylcholinesterase, butyrylcholinesterase, α-glucosidase, hyaluronidase and tyrosinase, as well as antioxidant and antimicrobial tests (**Figure 3**) At the lowest tested concentration (5 mg/mL), only the chloroform extract showed moderate hyaluronidase inhibitory activity (47%), while the methanol and *n*-hexane extracts exhibited weak (4%) or no inhibition. Notably, this activity at 5 mg/mL approaches the reference escin’s activity (IC_50_ = 7.35 ± 0.31 mg/mL), indicating comparable or potentially greater potency of the chloroform extract. Moreover, at higher concentrations (10 and 20 mg/mL), both the chloroform and methanol extracts demonstrated notable inhibitory effects (79-97%), and the *n*-hexane extract showed moderate to strong inhibitory activities (58% and 89%). The hyaluronidase inhibitory properties of various mushroom species have been confirmed by several previous studies. The extent of the observed effect largely depends on the mushroom extract examined, the polarity of the solvent used during extraction, and whether the fruiting body or the mycelium of the mushroom was analyzed. According to the study by Martins et al. (2023), one of the most effective hyaluronidase-inhibiting species was *Pisolithus tinctorius* (95% inhibition at a concentration of 1 mg/mL). The extract of the widely cultivated *Agaricus bisporus* showed a more moderate effect (74% inhibition at a concentration of 10 mg/mL) (MaChado-Carvalho et al., 2023), which is comparable to the results obtained in the present study. A research by Sułkowska-Ziaja et al. (2021) demonstrated a dose dependent hyaluronidase inhibitory activity of mycelial extracts from *Laetiporus sulphureus* and *Trametes versicolor*. Based on the promising results obtained for the mushroom extracts we decided to evaluate the fungal isolates in order to identify the compounds responsible for the observed hyaluronidase inhibitory activity. Among the examined secondary metabolites compound 8 exerted the highest activity against hyaluronidase with 91% at 10 mg/mL (17.40 mM), while compound 6 displayed moderate inhibitory properties with 32% at 10 mg/mL (22.79 mM), with no or very weak activity for the rest of the compounds (**Figure 4**). This result is comparable to that of the reference escin possessing an IC50 of 7.35 mg/mL (6.49 mM), indicating that the compound may possess equal or even superior potency compared to the standard. Our study is the first to report on the antihyaluronidase properties of ergosterol peroxide glucoside (8) and betulin (6). Previous studies highlighted the hyaluronidase inhibitor potential of natural pentacyclic triterpenes and synthetic betulinic acid derivatives (IC50 of 18.3 to 33.4 µM) as well (Luo et al., 2022). Hyaluronidase inhibitors have significant therapeutic potential due to their ability to regulate the degradation of hyaluronic acid, which is a key component of the extracellular matrix. Excessive hyaluronidase activity is implicated in a range of pathological processes; therefore, its inhibition may be relevant across multiple disease contexts including dermatological disorders, inflammatory diseases, and cancer.

The anticholinesterase assays carried out revealed that there is an important distinction between the AChE and BuChE inhibitory activities of *P. biformis*. While the methanol and chloroform extracts demonstrated noteworthy BuChE inhibitory activity at concentration of 40 mg/mL (51.42% and 50.78%) they did not show any inhibitory activity against acetylcholinesterase, and therefore the isolated individual metabolites were not further tested for AChE inhibition. The available data in the literature regarding the cholinesterase inhibitory activity of mushrooms underlines their potential in this field. The inhibitory effects of mushroom extracts against the two enzymes are highly variable: some extracts are effective against only one of the enzymes, while others act on both. Among the most effective species are the polypore *Phellinus hartigii* (Ünal et al., 2025), the well-known edible and medicinal mushroom *Hericium erinaceus* (Sevindik et al., 2024), and the underexplored edible *Tricholosporum goniospermum* (Angelini et al., 2020). In the latter case, no acetylcholinesterase inhibitory activity was observed, whereas extracts of *H. erinaceus* showed inhibitory effects against both enzymes. Regarding the BuChE inhibitory potential of our isolated constituents, the highest activity was observed for hydroxydihydrobovolide (3) with 56.94% inhibitory activity at 20mg/ml (100.87 mM), whereas the other compounds proved to be weaker BuChE inhibitors at tested concentrations. According to the literature there are only few secondary metabolites from mushrooms with notable BuChE inhibitory property, including triterpenes of the highly prized *Inonotus obliquus*, also known as Chaga (inhibitory activity of 2.40 to 28.72 μM) (Wei et al., 2022) and oleanonic acid (67.40% inhibition at 100 µg/ml) identified in *Fuscoporia torulosa* (Deveci et al., 2019). Although the isolated constituents showed lower potency than galantamine hydrobromide (IC_50_ = 0.86 mM), their activity remains of interest given the multi−target nature of biological activity and the potential safety advantages of natural products. These findings identify the *P. biformis* compounds as promising leads, though further studies are required to fully assess their therapeutic relevance. BuChE plays an important role as a compensating enzyme in the progression of Alzheimer’s disease. Research has demonstrated a strong association between BuChE activity and abnormal β-amyloid accumulation. Several studies propose that inhibiting BuChE could be a promising therapeutic approach for managing advanced stages of Alzheimer’s disease (Zhou and Huang, 2022).

The ABTS assay performed on three different concentrations (5, 10, and 20 mg/mL) highlight the antioxidant potential for different extracts of *P. biformis* depending on the concentration. At the lowest concentration (5 mg/mL) chloroform extract possessed moderate, while the methanol extract exerted weak antioxidant properties, with no activity for *n*-hexane extract. The higher concentrations (10 and 20 mg/mL) of methanol and chloroform extracts demonstrated moderate antioxidant properties, while the more concentrated hexane extract displayed a weak antioxidant activity (**Figure 3**). Despite the observable ABTS scavenging effect, the activity was significantly weaker than that of vitamin C (IC_50_ = 0.12 ± 0.01 mg/mL), a well−established potent antioxidant. The assessment of antioxidant capacity has received considerable attention in recent decades due to its nutritional relevance. Mushrooms, as functional foods, represent important components of a balanced and diverse healthy diet. The ABTS assay is a widely applied method for evaluating the antioxidant properties of both hydrophilic and lipophilic samples. Numerous studies have reported the antioxidant capacity of mushroom extracts, purified fractions, and isolated compounds using this method. Among the most potent species are the nutritionally significant *Pleurotus* species, including *P. sajor-caju*, *P. columbinus*, and *P. ostreatus* (IC_50_ values of 11–17 µg/mL), as well as *Agaricus bisporus* (IC_50_ = 30 µg/mL) (Elhusseiny et al., 2021).

Tyrosinase inhibitory assays revealed that the extracts of *P. biformis* possess the strongest inhibitory properties, with higher potential of chloroform (44.51%), showing activity that, while lower than that of azelaic acid (IC_50_ = 7.67 ± 0.92 mg/mL), approaches the potency of this clinically used reference inhibitor. Lower activity was noted for the methanol (34.26%), and *n*-hexane extract (**Figure 3**). According to the literature, certain mushroom species also exhibit significant tyrosinase inhibitory activity, including the split gill mushroom (*Schizophyllum commune*) (Arnamwong et al., 2024) and the edible *Tricholosporum goniospermum* (Angelini et al., 2020). However, in the latter case, extracts of varying polarity show substantial differences in activity, with the ethyl acetate extract demonstrating the highest efficacy. The activity values obtained in the present study are consistent with those reported for extracts and isolated components of the crust fungus, *Xylobolus subpileatus* (Felegyi et al., 2024).

Weaker inhibitory properties compared to acarbose (IC_50_ = 15.54 ± 1.49 mg/mL) were observed on α-glucosidase activity with slightly increased values for methanol and chloroform extracts, and the lowest activity for the *n*-hexane extract. According to our results the fungal extracts did not demonstrate antibacterial properties on any of the selected seven pathogen bacterial strains (MIC values >400 µg/mL). With respect to the bioactivity potential of *P. biforme*, it can be concluded that direct species-level comparisons remain limited due to the scarcity of studies addressing enzyme inhibitory activity within *Trichaptum*/*Pallidohirschioporus* species. Moreover, variations in assay design can substantially influence reported activity values; therefore, direct quantitative comparisons across studies are inherently constrained. Accordingly, interpretation should emphasize general activity trends rather than absolute differences in potency.

In conclusion, the comprehensive chemical analysis of *P. biformis* revealed the chemical and bioactivity potential of this polypore species. Subsequent use of combined chromatographic methods led to the isolation of eight compounds, among them a new butanolide natural product, named biformolide (1). However, the stereochemical assignment of biformolide (1) remains incomplete, and its absolute configuration warrants further investigation. Three other butanolide derivatives (2-4), as well as ergostane type steroids (7-8), the fatty acid derivative 5 and betulin (6) proved to be characteristic compounds of *P. biformis*. Several bioactivity assays have been performed which revealed the hyaluronidase and butyrylcholinesterase inhibitor and antioxidant properties of both extracts and isolated compounds of *P. biformis*. The results obtained recognize betulin (6), ergosterol peroxide glucoside (8) and hydroxydihydrobovolide (3) as the major bioactive compounds responsible for the observed hyaluronidase and butyrylcholinesterase inhibitor activity. While these findings highlight the biological potential of this polypore species, they should be regarded as results of a bioactivity screening, based on *in vitro* assays. Accordingly, further studies are required to fully validate the therapeutic relevance of *P. biformis* and its constituents.

## Data Availability

The datasets presented in this study can be found in online repositories. The names of the repository/repositories and accession number(s) can be found in the article/**Supplementary Material**.
