# Supplementary material for: Butenolides and triterpenoids from the fungus Pallidohirschioporus biformis (syn. Trichaptum biforme): isolation, structure determination and bioactivity profile
Source: Front Fungal Biol. 2026 Jun 3;7:1737900. doi: 10.3389/ffunb.2026.1737900 (PMC13292771; doi:10.3389/ffunb.2026.1737900)
Supplement: Supplementary file 1 [file SupplementaryFile1.docx]

Supplementary Material

Table of Contents

[1. HR-MS data of the isolated compounds 4](#_Toc212607370)

[Supplementary Table 1. HR-MS data of the isolated compounds 4](#_Toc212607371)

[2. Spectra and spectral data on biformolide (Compound 1) 6](#_Toc212607372)

[Supplementary Figure 1. HRESI-MS spectrum of compound 1 in negative ionization mode 6](#_Toc212607373)

[Supplementary Figure 2. MS-MS spectrum of compound 1 in negative ionization mode 6](#_Toc212607374)

[Supplementary Figure 3. HRESI-MS spectrum of compound 1 in positive ionization mode 7](#_Toc212607375)

[Supplementary Figure 4. MS-MS spectrum of compound 1 in positive ionization mode 7](#_Toc212607376)

[Supplementary Figure 5. ^1^H NMR spectrum of compound 1 8](#_Toc212607377)

[Supplementary Figure 6. ^13^C NMR spectrum (DEPTq) of compound 1 9](#_Toc212607378)

[Supplementary Figure 7. DEPT-edited HSQC spectrum of compound 1 10](#_Toc212607379)

[Supplementary Figure 8. HMBC spectrum of compound 1 10](#_Toc212607380)

[3. Spectra and spectral data on 9-(2-methoxy-3,4-dimethyl-5-oxofuran-2-yl)nonanoic acid (Compound 2) 11](#_Toc212607381)

[Supplementary Figure 9. HRESI-MS spectrum of compound 2 in negative ionization mode 11](#_Toc212607382)

[Supplementary Figure 10. MS-MS spectrum of compound 2 in negative ionization mode 11](#_Toc212607383)

[Supplementary Figure 11. HRESI-MS spectrum of compound 2 in positive ionization mode 12](#_Toc212607384)

[Supplementary Figure 12. MS-MS spectrum of compound 2 in positive ionization mode 12](#_Toc212607385)

[Supplementary Figure 13. ^1^H NMR spectrum of compound 2 13](#_Toc212607386)

[Supplementary Figure 14. ^1^H NMR spectrum of compound 2 with integrals 14](#_Toc212607387)

[Supplementary Figure 15. DEPT-edited HSQC spectrum of compound 2 15](#_Toc212607388)

[Supplementary Figure 16. HMBC spectrum of compound 2 15](#_Toc212607389)

[4. Spectra and spectral data on hydroxydihydrobovolide (Compound 3) 16](#_Toc212607390)

[Supplementary Figure 17. HRESI-MS spectrum of compound 3 in negative ionization mode 16](#_Toc212607391)

[Supplementary Figure 18. MS-MS spectrum of compound 3 in negative ionization mode 16](#_Toc212607392)

[Supplementary Figure 19. HRESI-MS spectrum of compound 3 in positive ionization mode 17](#_Toc212607393)

[Supplementary Figure 20. MS-MS spectrum of compound 3 in positive ionization mode 17](#_Toc212607394)

[Supplementary Figure 21. ^1^H NMR spectrum of compound 3 18](#_Toc212607395)

[Supplementary Figure 22. ^13^C NMR spectrum (DEPTq) of compound 3 19](#_Toc212607396)

[Supplementary Figure 23. DEPT-edited HSQC spectrum of compound 3 19](#_Toc212607397)

[Supplementary Figure 24. HMBC spectrum of compound 3 20](#_Toc212607398)

[5. Spectra and spectral data on Schumannione (Compound 4) 21](#_Toc212607399)

[Supplementary Figure 25. HRESI-MS spectrum of compound 4 in negative ionization mode 21](#_Toc212607400)

[Supplementary Figure 26. MS-MS spectrum of compound 4 in negative ionization mode 21](#_Toc212607401)

[Supplementary Figure 27. HRESI-MS spectrum of compound 4 in positive ionization mode 22](#_Toc212607402)

[Supplementary Figure 28. MS-MS spectrum of compound 4 in positive ionization mode 22](#_Toc212607403)

[Supplementary Figure 29. ^1^H NMR spectrum of compound 4 23](#_Toc212607404)

[Supplementary Figure 30. ^13^C NMR spectrum of compound 4 24](#_Toc212607405)

[Supplementary Figure 31. DEPT-edited HSQC spectrum of compound 4 25](#_Toc212607406)

[Supplementary Figure 32. HMBC spectrum of compound 4 25](#_Toc212607407)

[6. Spectra and spectral data on trans-4-hydroxy-2-nonenoic acid (Compound 5) 26](#_Toc212607408)

[Supplementary Figure 33. HRESI-MS spectrum of compound 5 in negative ionization mode 26](#_Toc212607409)

[Supplementary Figure 34. MS-MS spectrum of compound 5 in negative ionization mode 26](#_Toc212607410)

[Supplementary Figure 35. ^1^H NMR spectrum of compound 5 27](#_Toc212607411)

[Supplementary Figure 36. ^13^C NMR spectrum (DEPTq) of compound 5 28](#_Toc212607412)

[Supplementary Figure 37. DEPT-edited HSQC spectrum of compound 5 29](#_Toc212607413)

[Supplementary Figure 38. HMBC spectrum of compound 5 29](#_Toc212607414)

[7. Spectra and spectral data on betulin (Compound 6) 30](#_Toc212607415)

[Supplementary Figure 39. HRESI-MS spectrum of compound 6 in positive ionization mode 30](#_Toc212607416)

[Supplementary Figure 40. MS-MS spectrum of compound 6 in positive ionization mode 30](#_Toc212607417)

[Supplementary Figure 41. ^1^H NMR spectrum of compound 6 31](#_Toc212607418)

[Supplementary Figure 42. ^13^C NMR spectrum (DEPTq) of compound 6 32](#_Toc212607419)

[Supplementary Figure 43. COSY spectrum of compound 6 33](#_Toc212607420)

[Supplementary Figure 44. NOESY spectrum of compound 6 33](#_Toc212607421)

[Supplementary Figure 45. DEPT-edited HSQC spectrum of compound 6 34](#_Toc212607422)

[Supplementary Figure 46. HMBC spectrum of compound 6 34](#_Toc212607423)

[8. Spectra and spectral data on ergosterol peroxide (Compound 7) 35](#_Toc212607424)

[Supplementary Figure 47. HRESI-MS spectrum of compound 7 in positive ionization mode 35](#_Toc212607425)

[Supplementary Figure 48. MS-MS spectrum of compound 7 in positive ionization mode 35](#_Toc212607426)

[Supplementary Figure 49. ^1^H NMR spectrum of compound 7 36](#_Toc212607427)

[Supplementary Figure 50. ^13^C NMR spectrum (DEPTq) of compound 7 37](#_Toc212607428)

[Supplementary Figure 51. COSY spectrum of compound 7 37](#_Toc212607429)

[Supplementary Figure 52. 2D ROESY spectrum of compound 7 38](#_Toc212607430)

[Supplementary Figure 53. DEPT-edited HSQC spectrum of compound 7 38](#_Toc212607431)

[Supplementary Figure 54. HMBC spectrum of compound 7 39](#_Toc212607432)

[9. Spectra and spectral data on ergosterol peroxide glucoside (Compound 8) 40](#_Toc212607433)

[Supplementary Figure 55. HRESI-MS spectrum of compound 8 in positive ionization mode 40](#_Toc212607434)

[Supplementary Figure 56. MS-MS spectrum of compound 8 in positive ionization mode 40](#_Toc212607435)

[Supplementary Figure 57. ^1^H NMR spectrum of compound 8 41](#_Toc212607436)

[Supplementary Figure 58. ^13^C NMR spectrum (DEPTq) of compound 8 42](#_Toc212607437)

[Supplementary Figure 59. COSY spectrum of compound 8 43](#_Toc212607438)

[Supplementary Figure 60. NOESY spectrum of compound 8 43](#_Toc212607439)

[Supplementary Figure 61. DEPT-edited HSQC spectrum of compound 8 44](#_Toc212607440)

[Supplementary Figure 62. HMBC spectrum of compound 8 44](#_Toc212607441)

# HR-MS data of the isolated compounds

## Supplementary Table 1. HR-MS data of the isolated compounds

| **No.** | **Molecular formula** | **Ion Mode** | **Measured Mass**  **(*m/z*)** | **Error (ppm)** | **Fragment ions (*m/z*)** | **Structure** |
| --- | --- | --- | --- | --- | --- | --- |
| **1** | C_12_H_20_O_4_ | [M – H] ^–^ | 227.1286 | 3.5 | 209.1178 (C_12_H_17_O_3_) [M–H–H_2_O]^–^, 195.1014 (C_11_H_15_O_3_) [M–H–CH_3_OH] ^–^, 183.1382 (C_11_H_19_O_2_) [M–H–CO_2_] ^–^, 167.1064 (C_10_H_15_O_2_) [M–H–C_2_H_4_O_2_] ^–^, 151.1115 (C_10_H_15_O) | Biformolide (Compound **1**) |
|  |  | [M + H]^+^ | 229.1427 | 3.1 | 211.1322 (C_12_H_19_O_3_) [M+H–H_2_O]^+^, 183.1375 (C_11_H_19_O_2_) [M+H–CH_2_O_2_]^+^, 151.1111 (C_10_H_15_O) [M+H–CH_2_O_2_–CH_3_OH]^+^ |  |
| **2** | C_16_H_26_O_5_ | [M – H] ^–^ | 297.1708 | 3.9 | 265.1445 (C_15_H_21_O_4_) [M–H–CH_3_OH]^–^, 253.1808 (C_15_H_25_O_3_) [M–H–CO_2_]^–^, 239.1648 (C_14_H_23_O_3_) [M–H–C_2_H_4_O_2_]^–^, 221.1542 (C_14_H_21_O_2_) [M–H–CO_2_–CH_3_OH]^–^ | 9-(2-methoxy-3,4-dimethyl-5-oxofuran-2-yl)nonanoic acid  (Compound **2**) |
|  |  | [M + H]^+^ | 299.1843 | 3.5 | 267.1581 (C_15_H_23_O_4_) [M+H–CH_3_OH]^+^, 249.1478 (C_15_H_21_O_3_) [M+H–CH_3_OH–H_2_O]^+^, 231.1373 (C_15_H_19_O_2_) [M+H–CH_3_OH–2×H_2_O] ^+^, 221.1528 (C_14_H_21_O_2_) [M+H–CH_3_OH–CO_2_]^+^, 213.1266 (C_15_H_17_O) [M+H–CH_3_OH–3×H_2_O] ^+^, 203.1425 (C_14_H_19_O) [M+H–CH_3_OH–CO_2_–H_2_O]^+^ |  |
| **3** | C_11_H_18_O_3_ | [M – H] ^–^ | 197.1177 | 2.3 | 179.1068 (C_11_H_15_O_2_) [M–H–H_2_O]^–^, 153.1273 (C_10_H_17_O) [M–H–CO_2_]^–^ | Hydroxydihydrobovoli-de  (Compound **3**) |
|  |  | [M + H]^+^ | 199.1323 | 3.0 | 181.1217 (C_11_H_17_O_2_) [M+H–H_2_O]^+^, 163.1112 [M+H–2×H_2_O]^+^ (C_11_H_15_O), 153.1268 (C_10_H_17_O) [M+H–CH_2_O_2_]^+^, 145.1007 (C_11_H_13_) [M+H–3×H_2_O]^+^, 135.1164 (C_10_H_15_) [M+H–CO_2_–H_2_O]^+^ |  |
| **4** | C_11_H_18_O_4_ | [M – H] ^–^ | 213.1127 | 2.5 | 195.1022 (C_11_H_15_O_3_) [M–H–H_2_O]^–^, 169.1223 (C_10_H_17_O_2_) [M–H–CO_2_]^–^, 151.1115 (C_10_H_15_O) [M–H–CO_2_–H_2_O]^–^ | Schumannione  (Compound **4**) |
|  |  | [M + H]^+^ | 215.1271 | 3.1 | 197.1166 (C_11_H_17_O_3_) [M+H–H_2_O]^+^, 169.1217 (C_10_H_17_O_2_) [M+H–CH_2_O_2_]^+^, 153.1269 (C_10_H_17_O) [M+H–H_2_O–CO_2_]^+^, 151.1115 (C_10_H_15_O) [M+H–H_2_O–CH_2_O_2_]^+^ |  |
| **5** | C_9_H_16_O_3_ | [M – H] ^–^ | 171.1017 | 0.9 | 153.0909 (C_9_H_13_O_2_) [M–H–H_2_O]^–^, 127.1115 (C_8_H_15_O) [M–H–CO_2_]^–^, 125.0958 (C_8_H_13_O) [M–H–CH_2_O_2_]^–^ | *trans*-4-hydroxy-2-nonenoic acid  (Compound **5**) |
| **6** | C_30_H_46_O_2_ | [M + H]^+^ | 443.3868 | 2.8 | 425.3764 (C_30_H_49_O) [M+H–H_2_O]^+^, 407.3660 (C_30_H_47_) [M+H–H_2_O]^+^ | Betulin  (Compound **6**) |
| **7** | C_28_H_44_O_3_ | [M + H]^+^ | 429.3359 | 1.0 | 395.3301 (C_28_H_43_O) [M+H–H_2_O_2_]^+^, 377.3196 (C_28_H_41_) [M+H–H_2_O–H_2_O_2_]^+^ | Ergosterol peroxide  (Compound **7**) |
| **8** | C_34_H_54_O_8_ | [M + H]^+^ | 591.3883 | 1.4 | 573.3779 (C_34_H_53_O_7_) [M+H–H_2_O]^+^, 555.3675 (C_34_H_51_O_6_) [M+H–2H_2_O]^+^, 429.3362 (C_28_H_45_O_3_) [M + H – Glc]^+^, 411.3255 (C_28_H_43_O) [M + H – Glc–H_2_O]^+^, 395.3266 (C_28_H_43_O) [M+H–Glc–H_2_O_2_]^+^, 377.3200 (C_28_H_41_) [M+H–Glc –H_2_O–H_2_O_2_]^+^ | Ergosterol peroxide glucoside  (Compound **8**) |

# Spectra and spectral data on biformolide (Compound 1)

HRESIMS *m/z* 227.1286 [M - H]- (Δ 3.5 ppm; C_12_H_19_O_4_); HRESI-MSMS (CID = 15%, 30%, 45%) *m/z* 209.1178, 195.1014, 183.1382, 167.1064, 151.1115.

HRESIMS *m/z* 229.1427 [M + H]+ (Δ 3.1 ppm; C_12_H_21_O_4_); HRESI-MSMS (CID = 15%, 30%, 45%) *m/z* 211.1322, 183.1375, 151.1111.

|  |
| --- |
| Supplementary Figure 1. HRESI-MS spectrum of compound 1 in negative ionization mode |

|  |
| --- |
| Supplementary Figure 2. MS-MS spectrum of compound 1 in negative ionization mode |

|  |
| --- |
| Supplementary Figure 3. HRESI-MS spectrum of compound 1 in positive ionization mode |

|  |
| --- |
| Supplementary Figure 4. MS-MS spectrum of compound 1 in positive ionization mode |

| 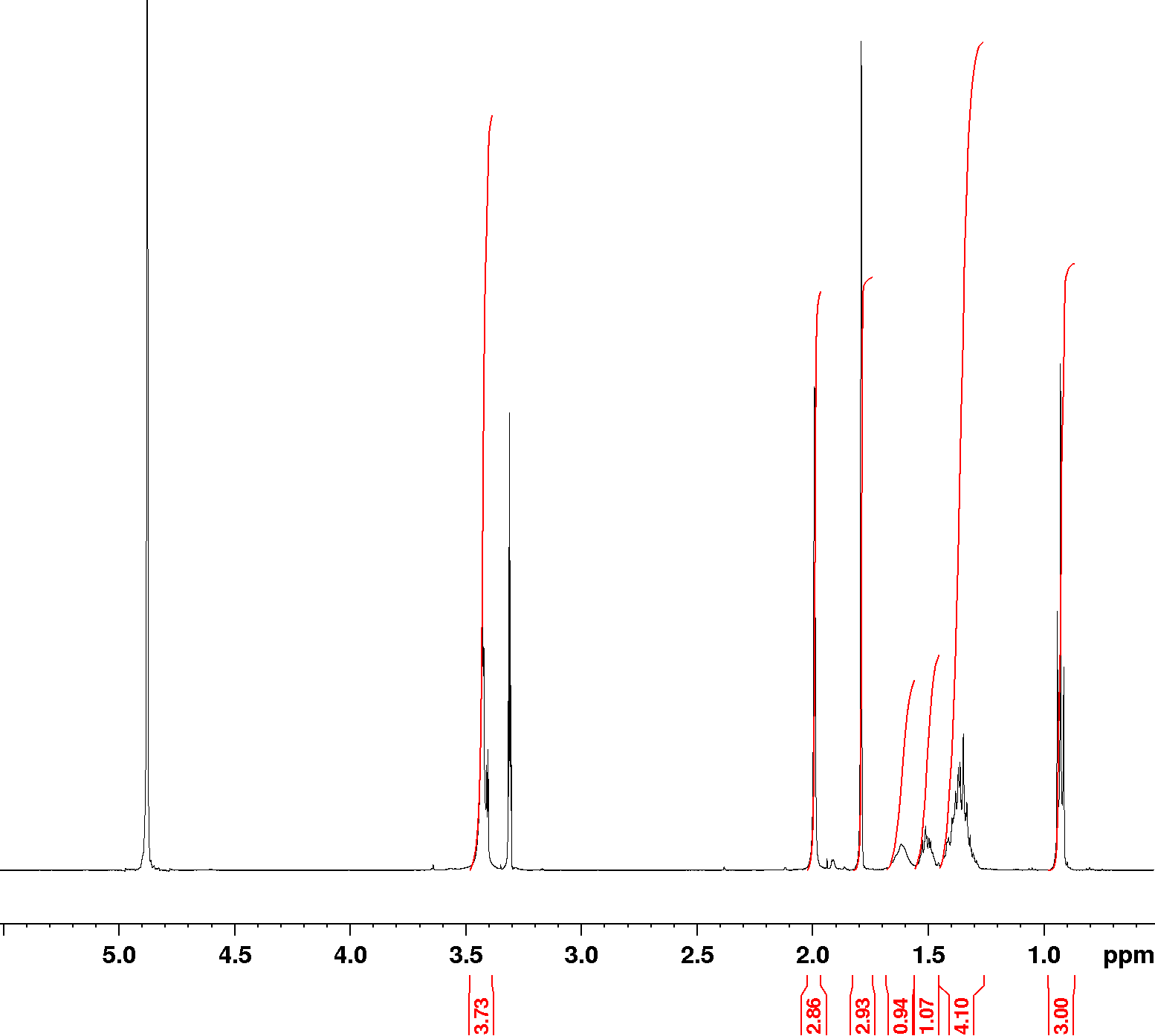 |
| --- |
| Supplementary Figure 5. ^1^H NMR spectrum of compound 1 |

| 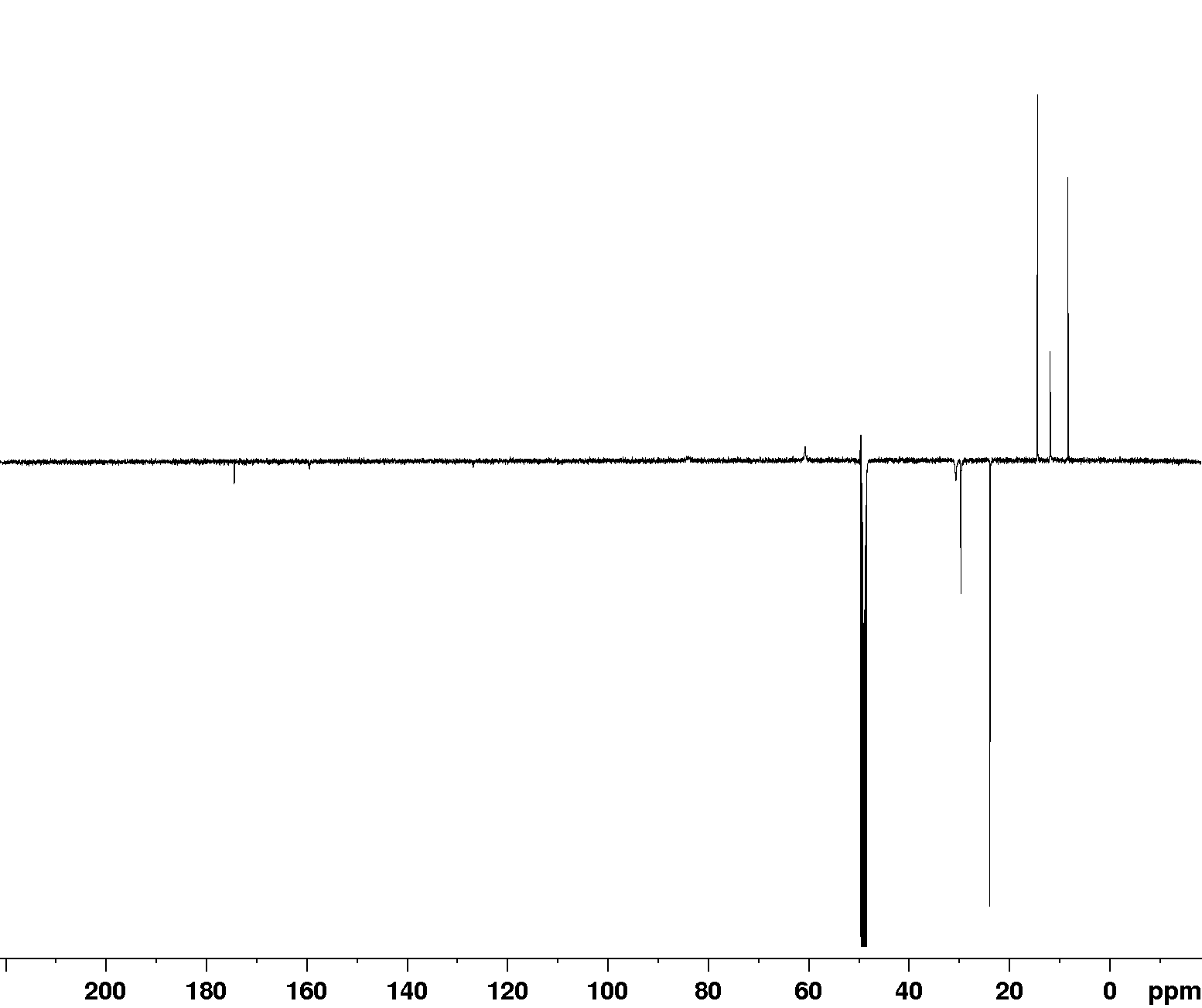 |
| --- |
| Supplementary Figure 6. ^13^C NMR spectrum (DEPTq) of compound 1 |

| 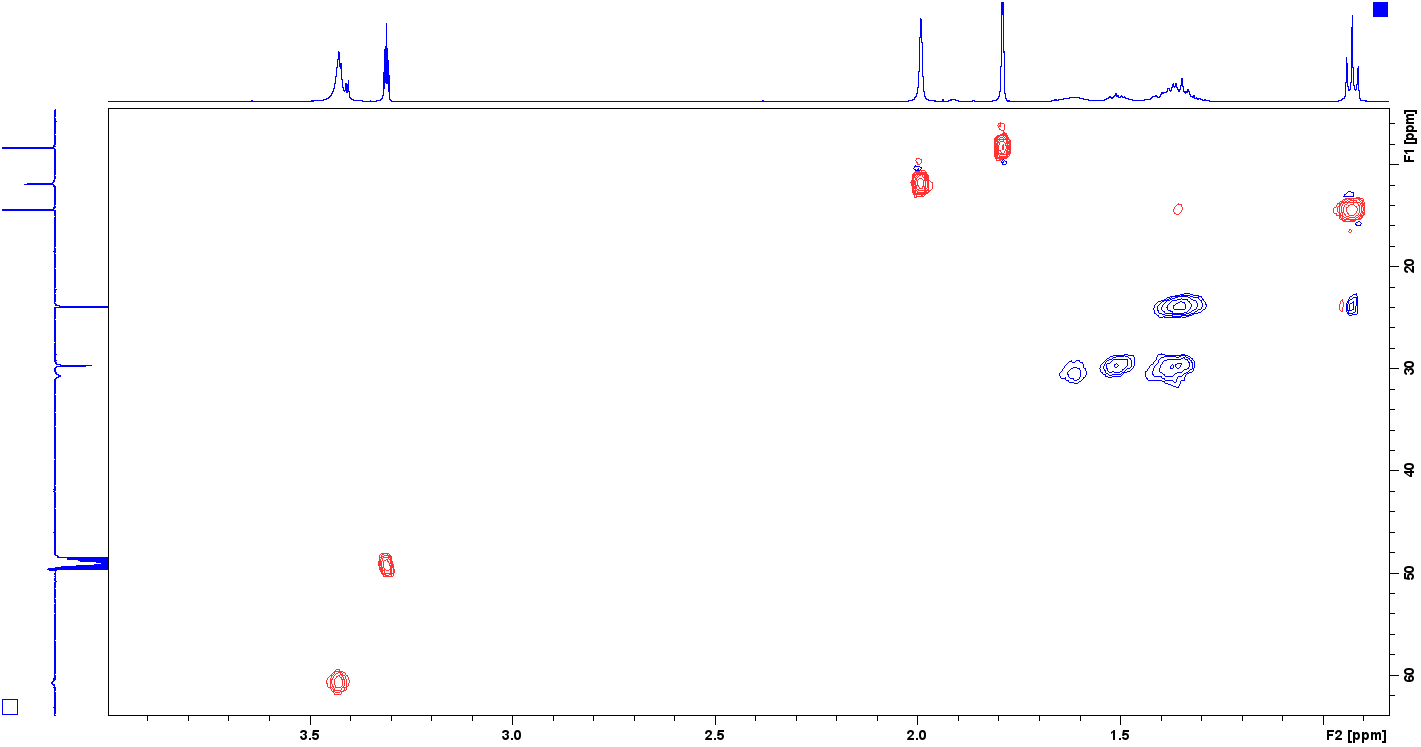 |
| --- |
| Supplementary Figure 7. DEPT-edited HSQC spectrum of compound 1 |

| 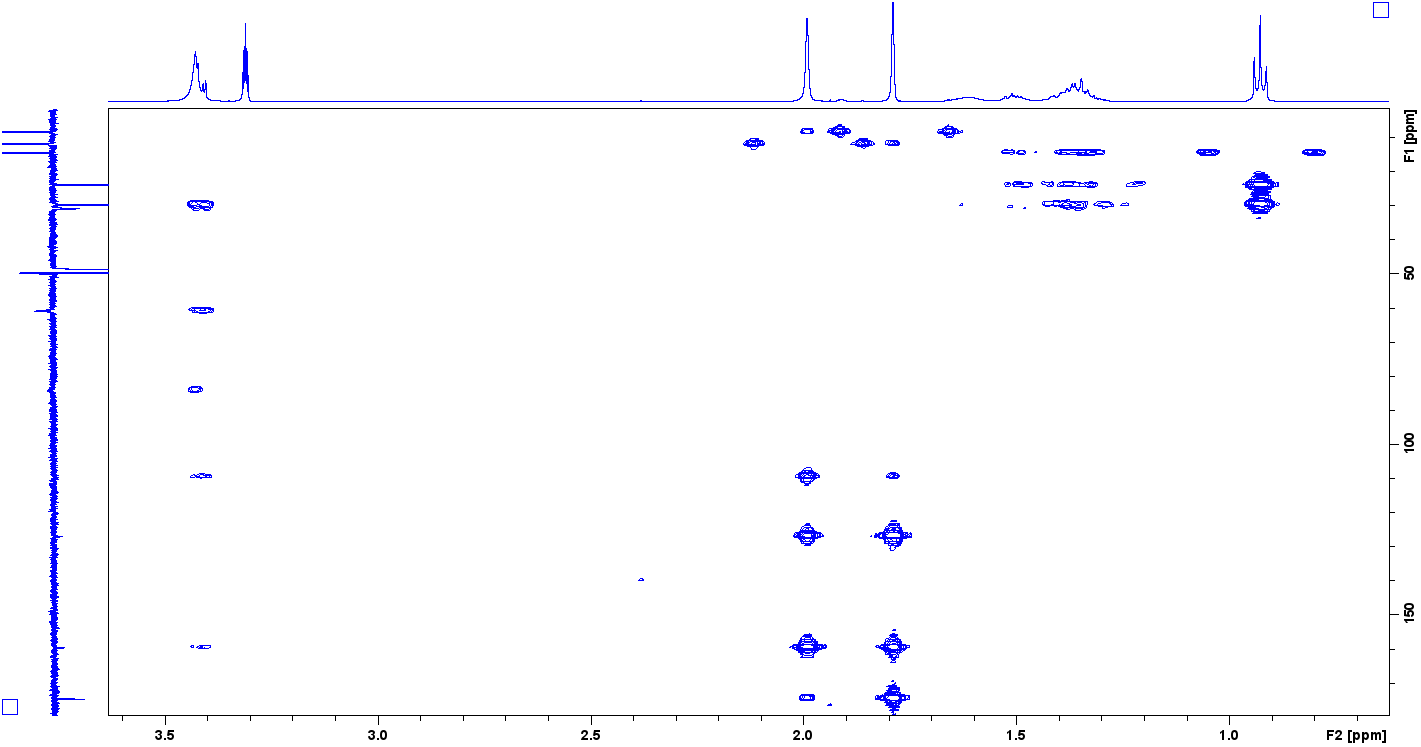 |
| --- |
| Supplementary Figure 8. HMBC spectrum of compound 1 |

# Spectra and spectral data on 9-(2-methoxy-3,4-dimethyl-5-oxofuran-2-yl)nonanoic acid (Compound 2)

HRESIMS *m/z* 297.1708 [M - H]- (Δ 3.9 ppm; C_16_H_25_O_5_); HRESI-MSMS (CID = 15%, 30%, 45%) *m/z* 283.1548, 265.1445, 253.1808, 239.1648, 221.1542.

HRESIMS *m/z* 299.1843 [M + H]+ (Δ 3.5 ppm; C_16_H_27_O_5_); HRESI-MSMS (CID = 15%, 30%, 45%) *m/z* 267.1581, 249.1478, 231.1373, 221.1528, 213.1266, 203.1425, 185.1319, 175.1476, 137.0594.

|  |
| --- |
| Supplementary Figure 9. HRESI-MS spectrum of compound 2 in negative ionization mode |

|  |
| --- |
| Supplementary Figure 10. MS-MS spectrum of compound 2 in negative ionization mode |

|  |
| --- |
| Supplementary Figure 11. HRESI-MS spectrum of compound 2 in positive ionization mode |

|  |
| --- |
| Supplementary Figure 12. MS-MS spectrum of compound 2 in positive ionization mode |

| 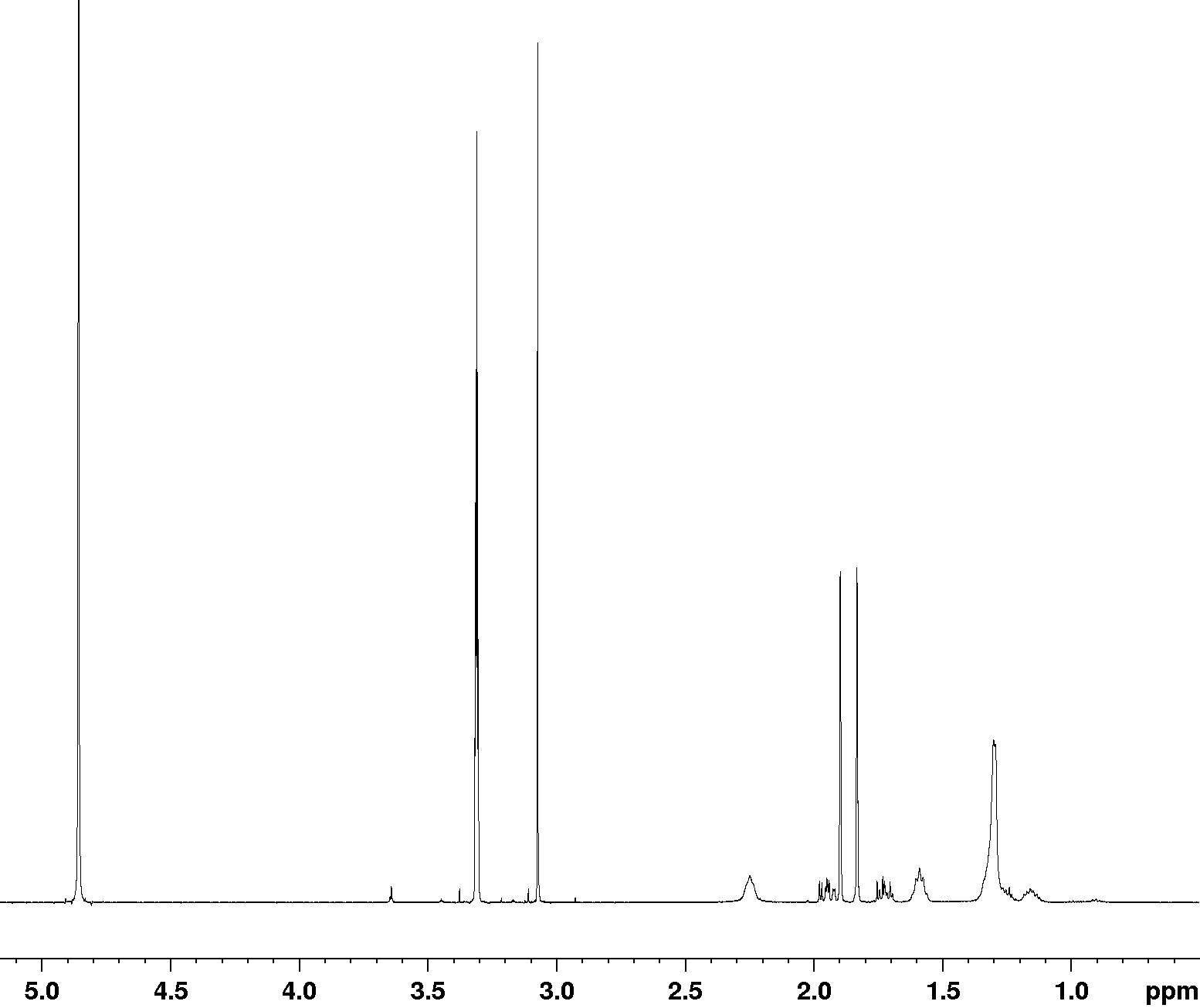 |
| --- |
| Supplementary Figure 13. ^1^H NMR spectrum of compound 2 |

| 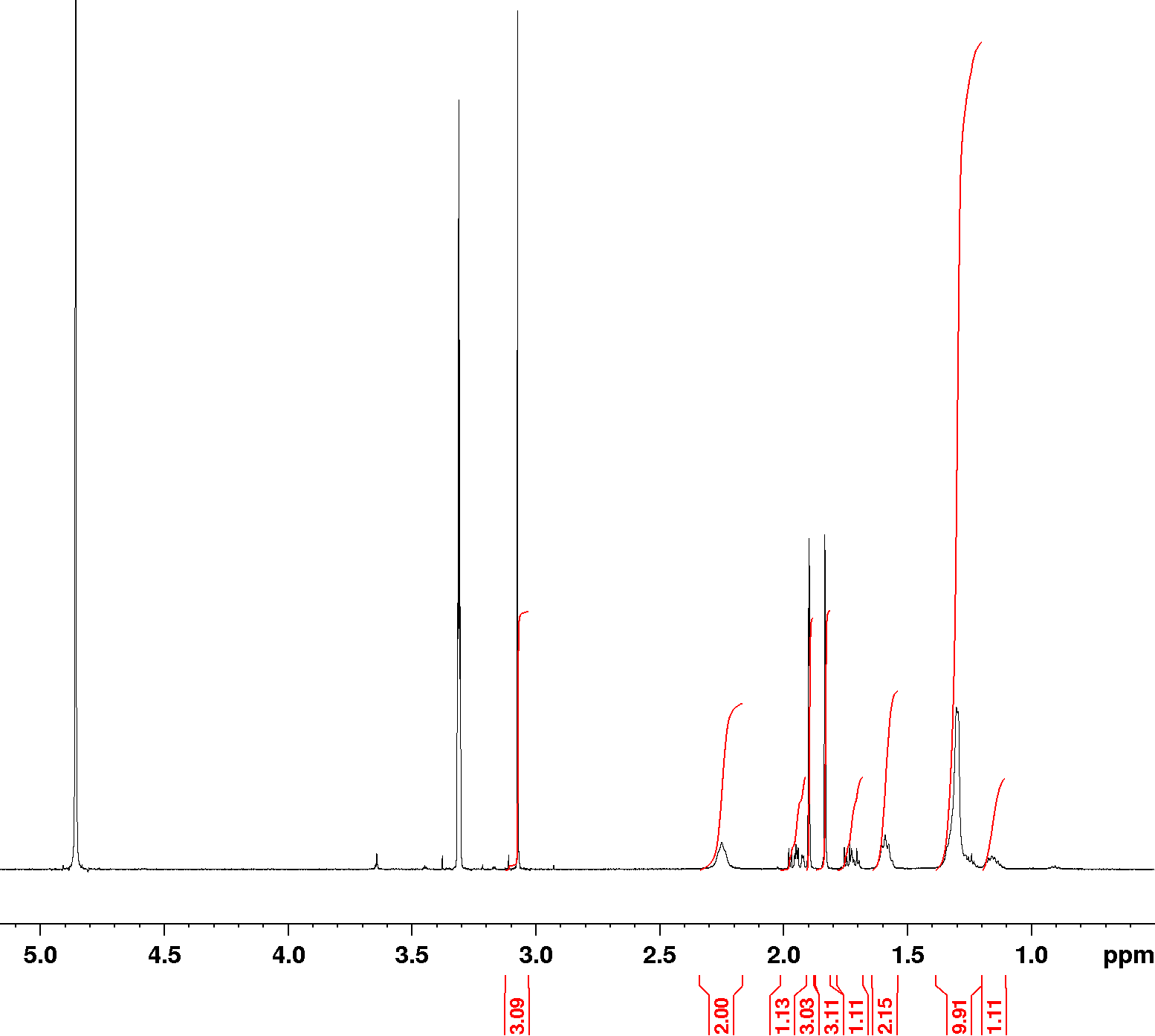 |
| --- |
| Supplementary Figure 14. ^1^H NMR spectrum of compound 2 with integrals |

| 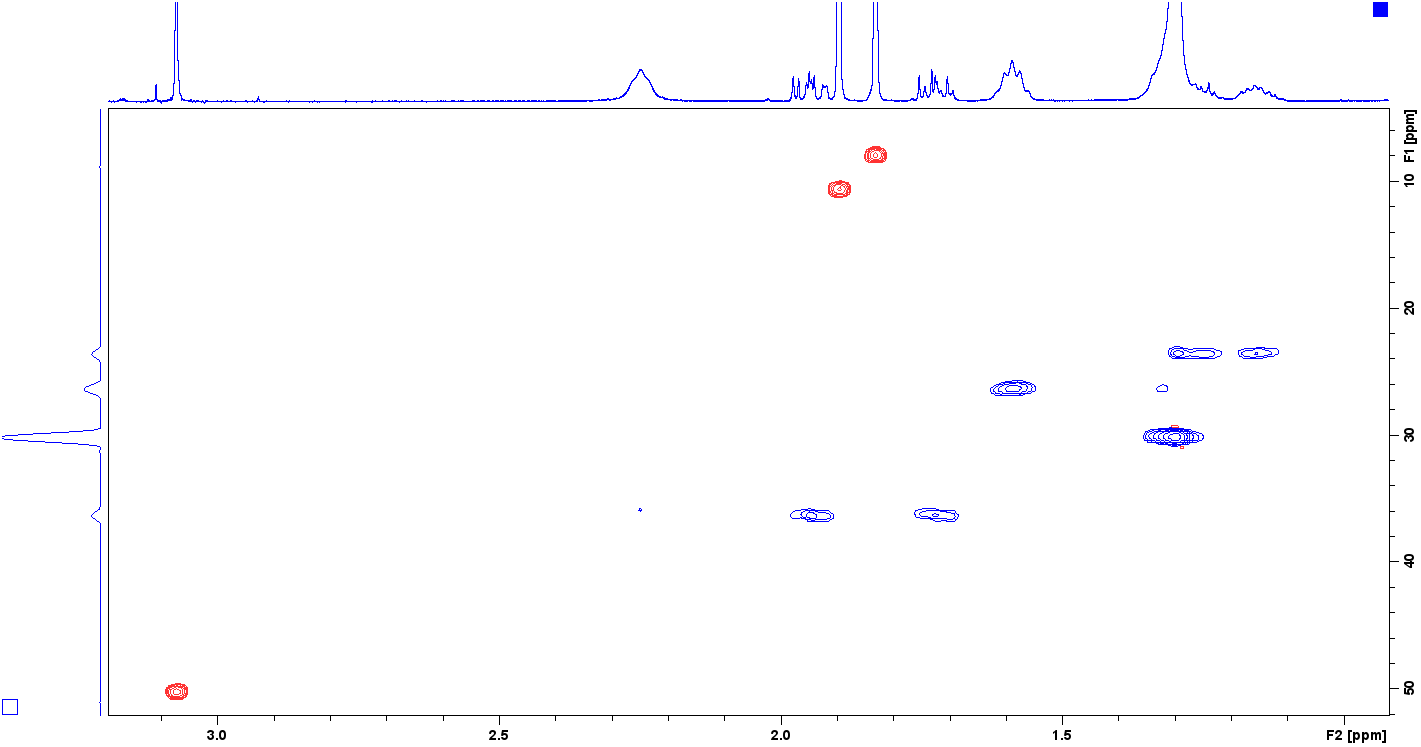 |
| --- |
| Supplementary Figure 15. DEPT-edited HSQC spectrum of compound 2 |

| 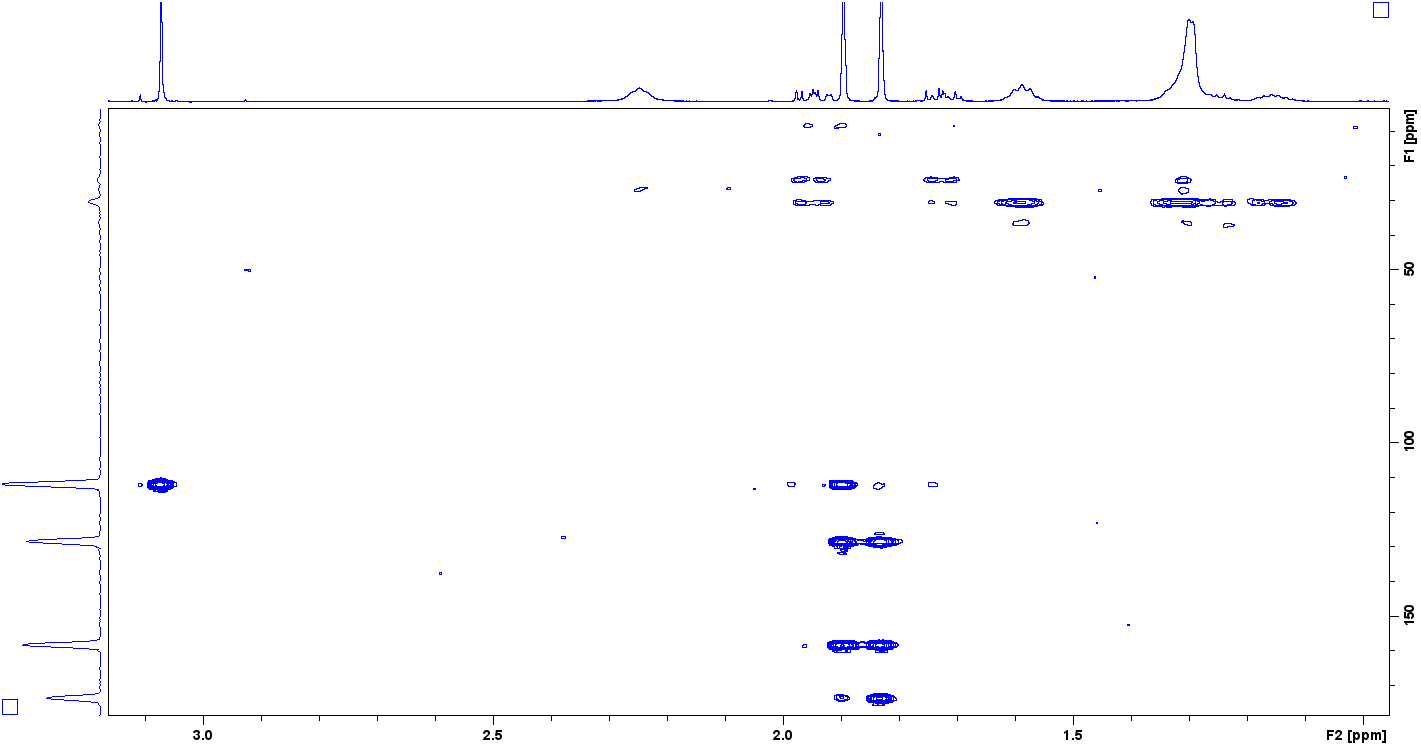 |
| --- |
| Supplementary Figure 16. HMBC spectrum of compound 2 |

# Spectra and spectral data on hydroxydihydrobovolide (Compound 3)

HRESIMS *m/z* 197.1177 [M - H]- (Δ 2.3 ppm; C_11_H_17_O_3_); HRESI-MSMS (CID = 15%, 30%, 45%) *m/z* 179.1068, 166.9926, 153.1273.

HRESIMS *m/z* 199.1323 [M + H]+ (Δ 3.0 ppm; C_11_H_19_O_3_); HRESI-MSMS (CID = 15%, 30%, 45%) *m/z* 181.1217, 163.1112; 153.1268, 145.1007, 139.0749, 135.1164, 127.0752, 125.0595.

| Supplementary Figure 17. HRESI-MS spectrum of compound 3 in negative ionization mode |
| --- |

|  |
| --- |
| Supplementary Figure 18. MS-MS spectrum of compound 3 in negative ionization mode |

|  |
| --- |
| Supplementary Figure 19. HRESI-MS spectrum of compound 3 in positive ionization mode |

|  |
| --- |
| Supplementary Figure 20. MS-MS spectrum of compound 3 in positive ionization mode |

| 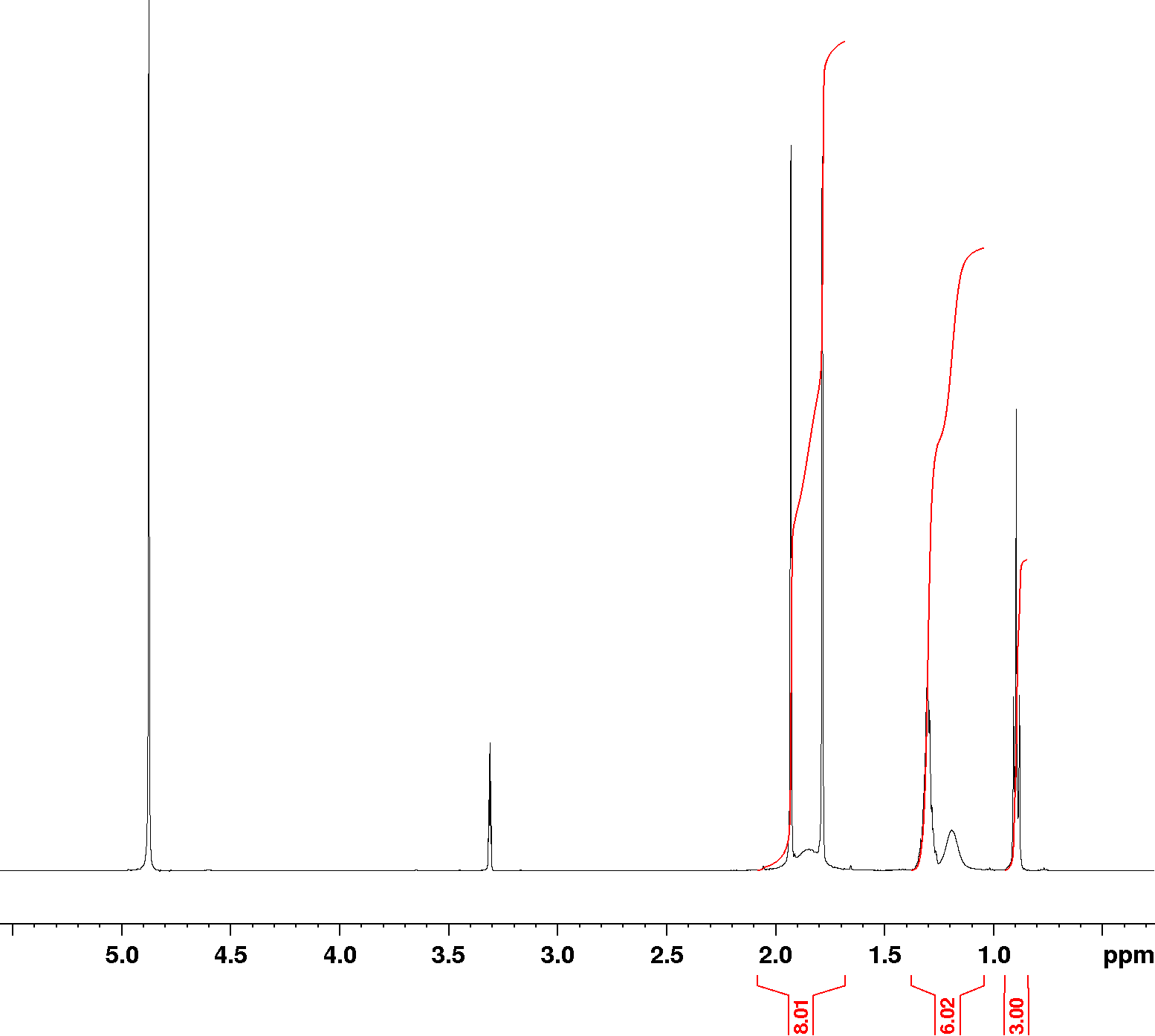 |
| --- |
| Supplementary Figure 21. ^1^H NMR spectrum of compound 3 |

| 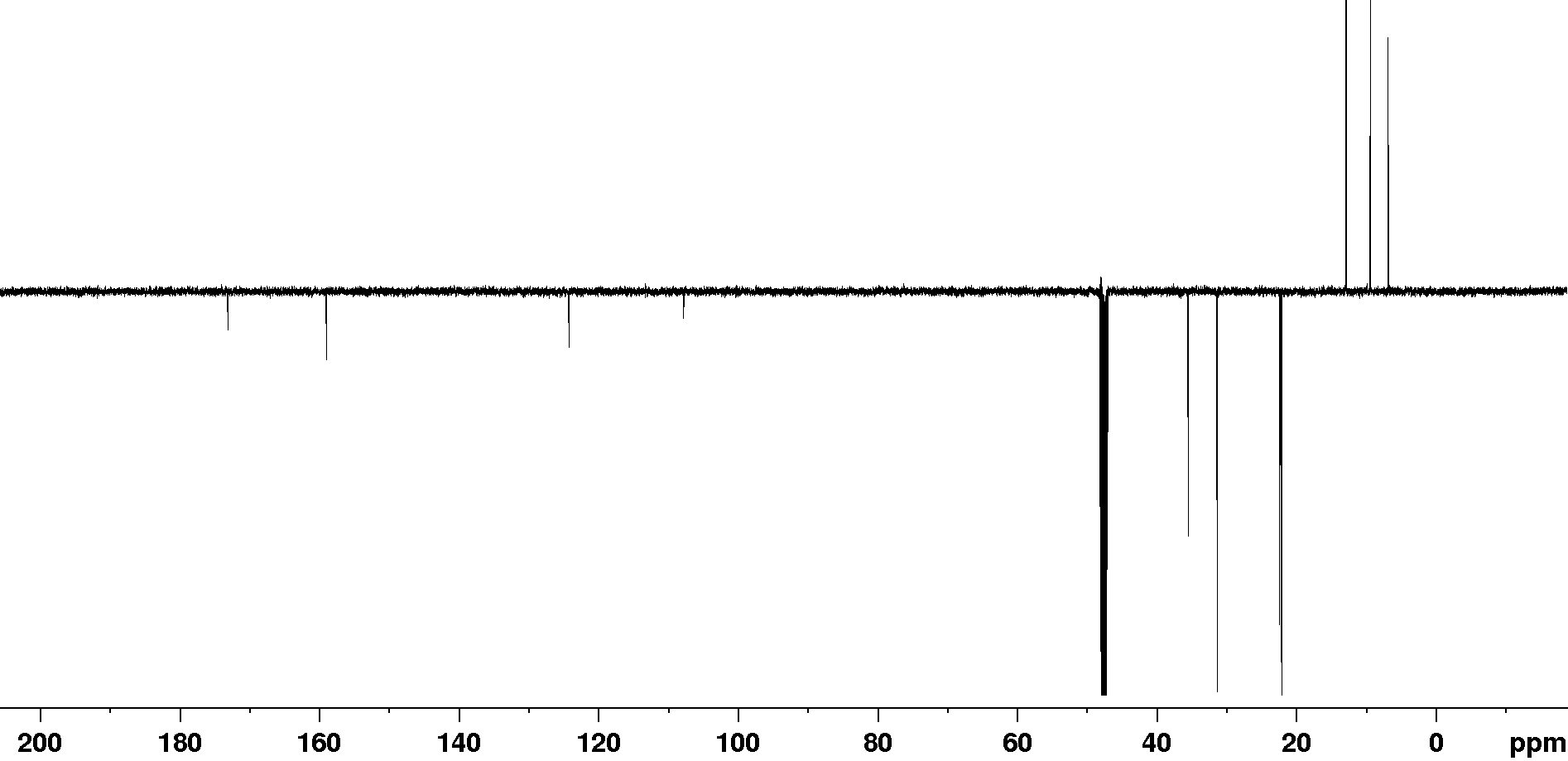 |
| --- |
| Supplementary Figure 22. ^13^C NMR spectrum (DEPTq) of compound 3 |

| 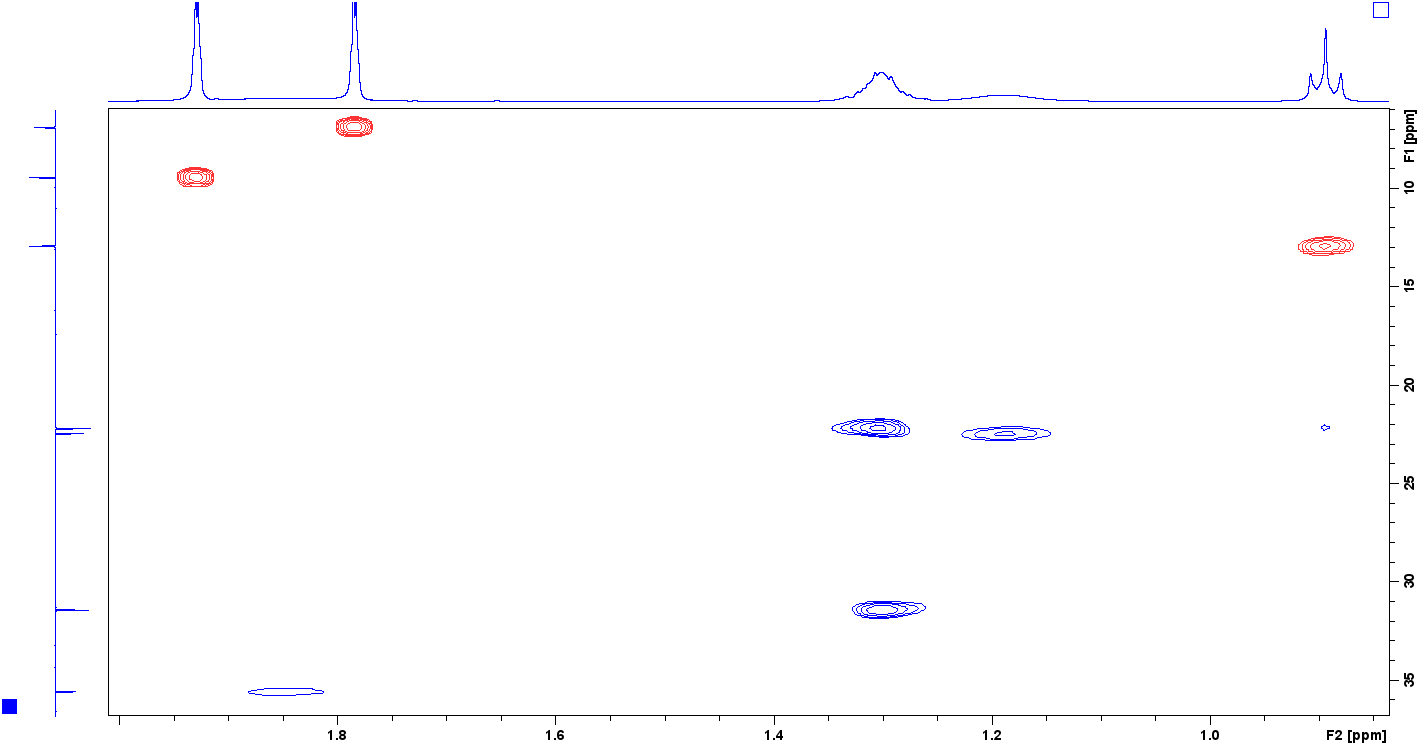 |
| --- |
| Supplementary Figure 23. DEPT-edited HSQC spectrum of compound 3 |

| 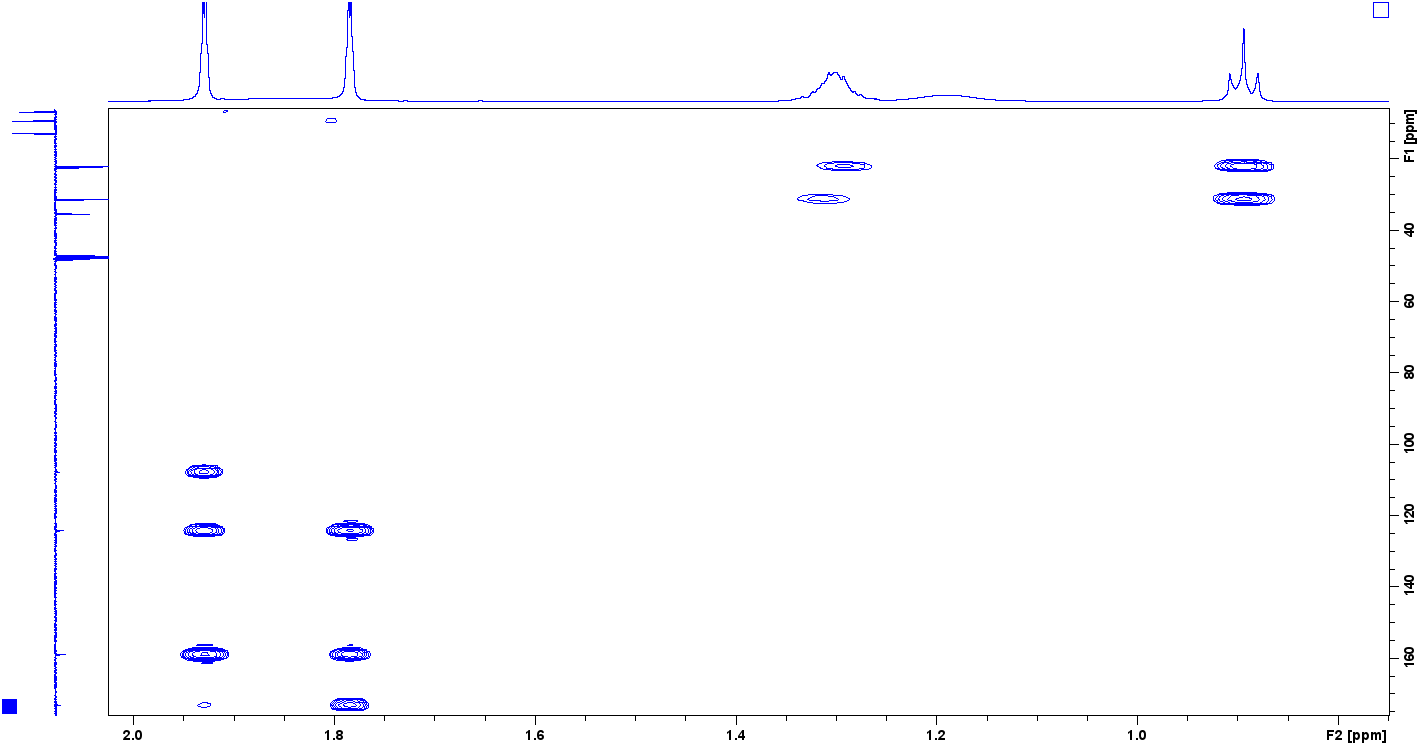 |
| --- |
| Supplementary Figure 24. HMBC spectrum of compound 3 |

# Spectra and spectral data on Schumannione (Compound 4)

HRESIMS *m/z* 213.1127 [M - H]- (Δ 2.5 ppm; C_11_H_17_O_4_); HRESI-MSMS (CID = 15%, 30%, 45%) *m/z* 195.1022, 169.1223, 151.1115

HRESIMS *m/z* 215.1271 [M + H]+ (Δ 3.1 ppm; C_11_H_19_O_4_); HRESI-MSMS (CID = 15%, 30%, 45%) *m/z* 197.1166, 169.1217, 153.1269.

|  |
| --- |
| Supplementary Figure 25. HRESI-MS spectrum of compound 4 in negative ionization mode |

|  |
| --- |
| Supplementary Figure 26. MS-MS spectrum of compound 4 in negative ionization mode |

|  |
| --- |
| Supplementary Figure 27. HRESI-MS spectrum of compound 4 in positive ionization mode |

|  |
| --- |
| Supplementary Figure 28. MS-MS spectrum of compound 4 in positive ionization mode |
| 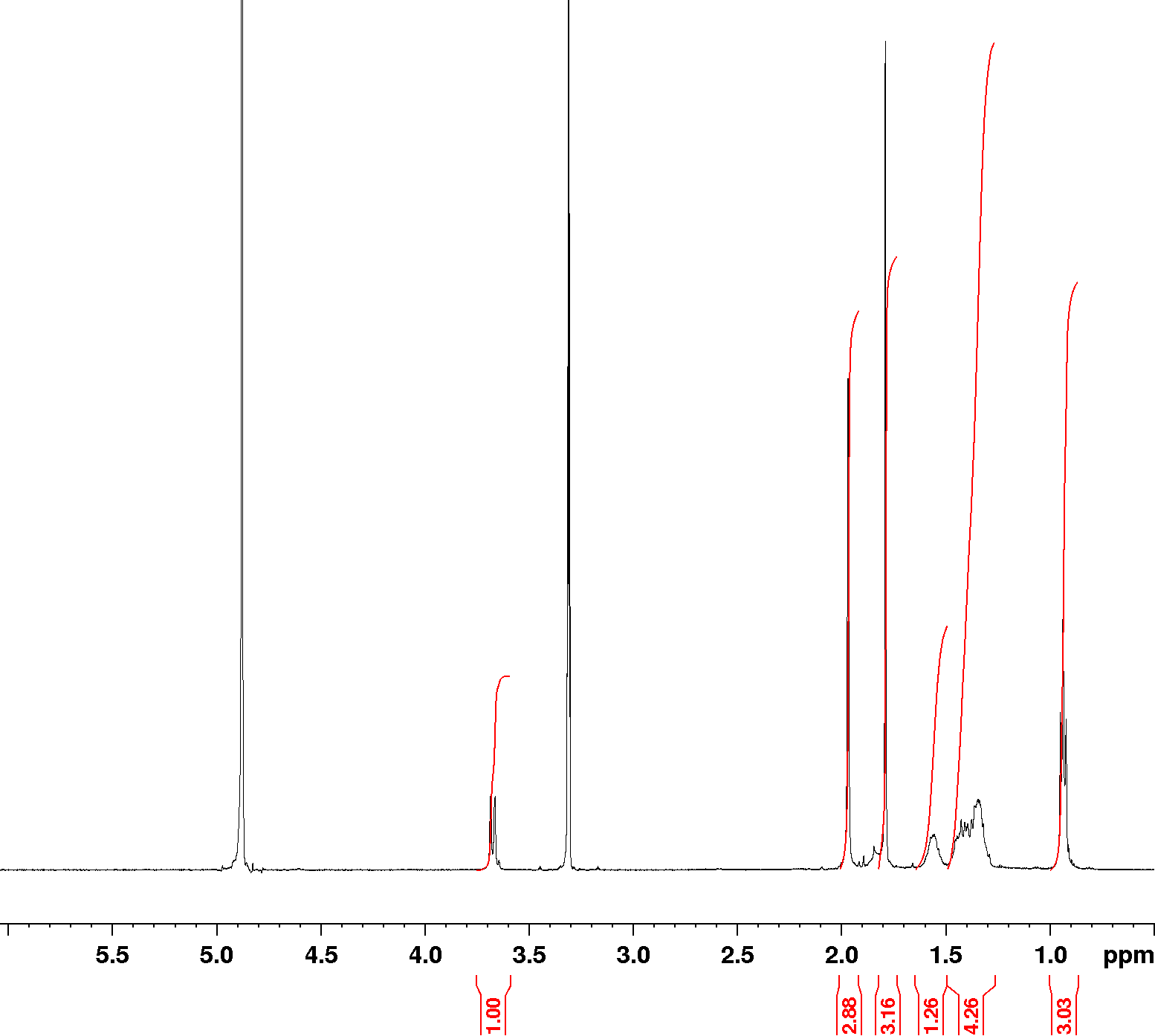 |
| Supplementary Figure 29. ^1^H NMR spectrum of compound 4 |

| 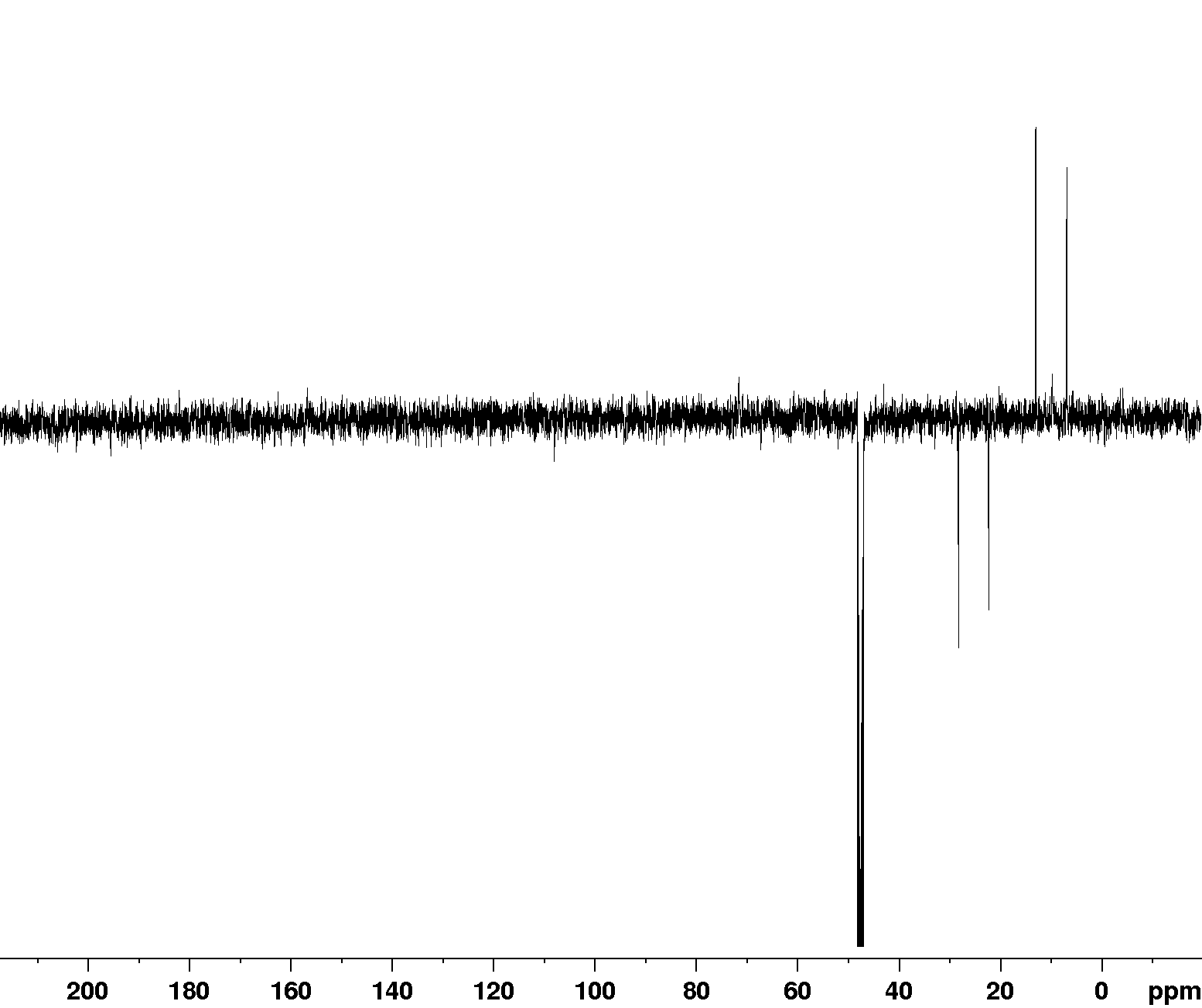 |
| --- |
| Supplementary Figure 30. ^13^C NMR spectrum of compound 4 |

| 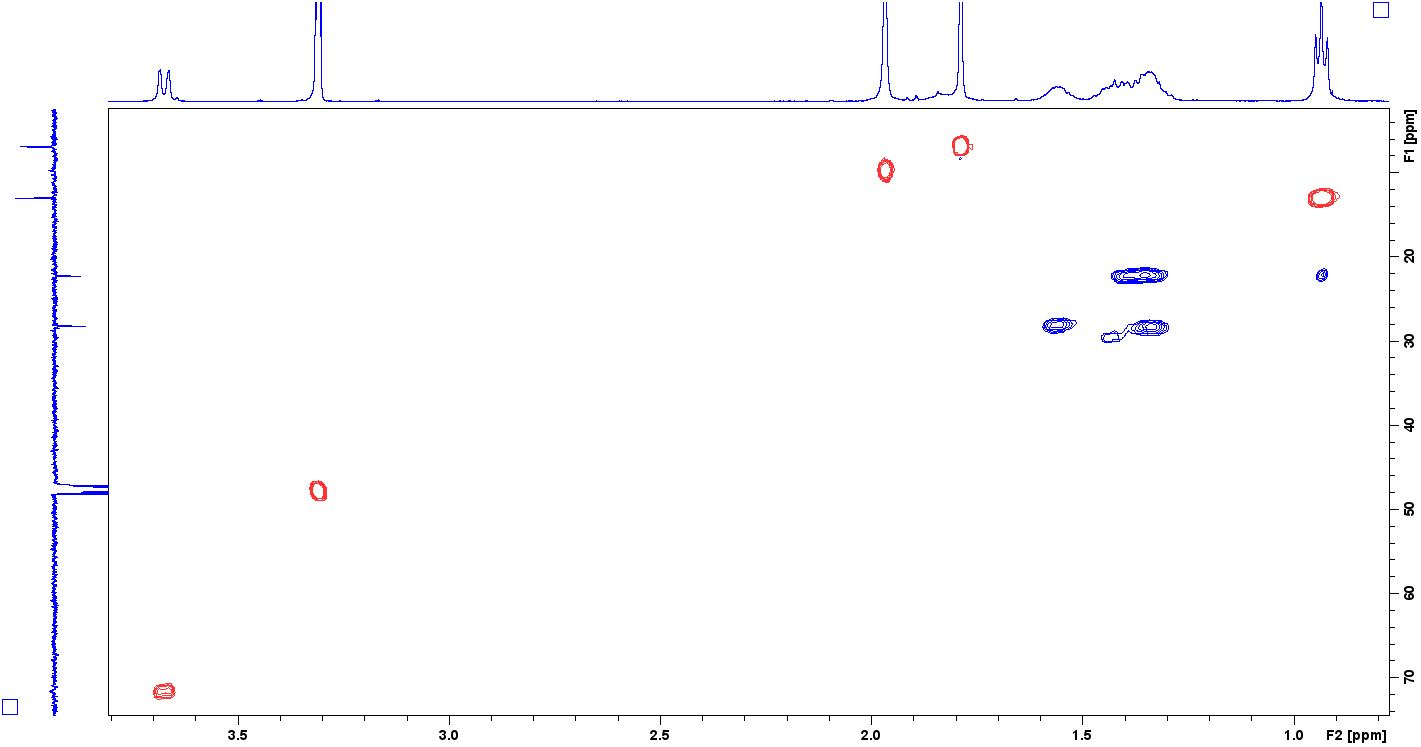 |
| --- |
| Supplementary Figure 31. DEPT-edited HSQC spectrum of compound 4 |

| 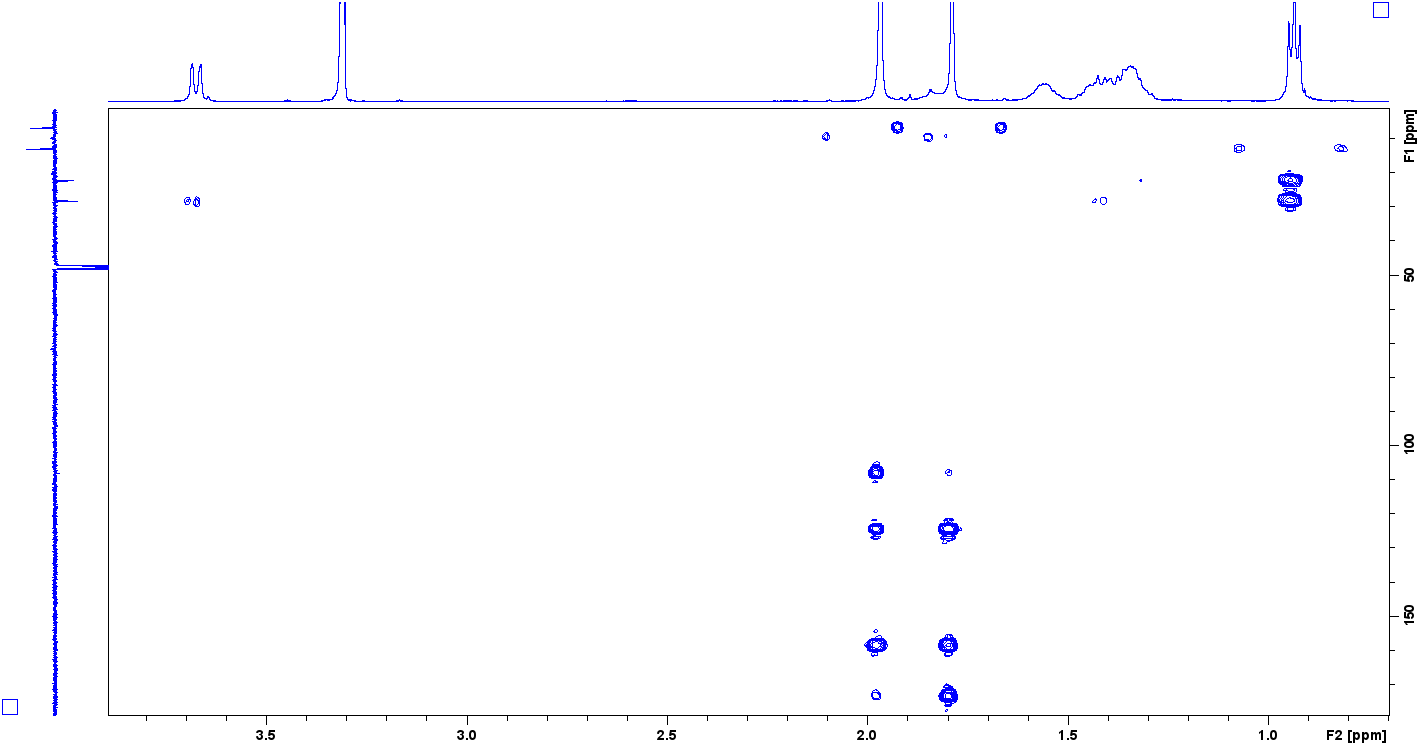 |
| --- |
| Supplementary Figure 32. HMBC spectrum of compound 4 |

# Spectra and spectral data on *trans*-4-hydroxy-2-nonenoic acid (Compound 5)

HRESIMS *m/z* 171.1017 [M - H]- (Δ 0.9 ppm; C_9_H_15_O_3_); HRESI-MSMS (CID = 15%, 30%, 45%) *m/z* 153.0909, 127.1115, 125.0958, 116.9271.

|  |
| --- |
| Supplementary Figure 33. HRESI-MS spectrum of compound 5 in negative ionization mode |

|  |
| --- |
| Supplementary Figure 34. MS-MS spectrum of compound 5 in negative ionization mode |

| 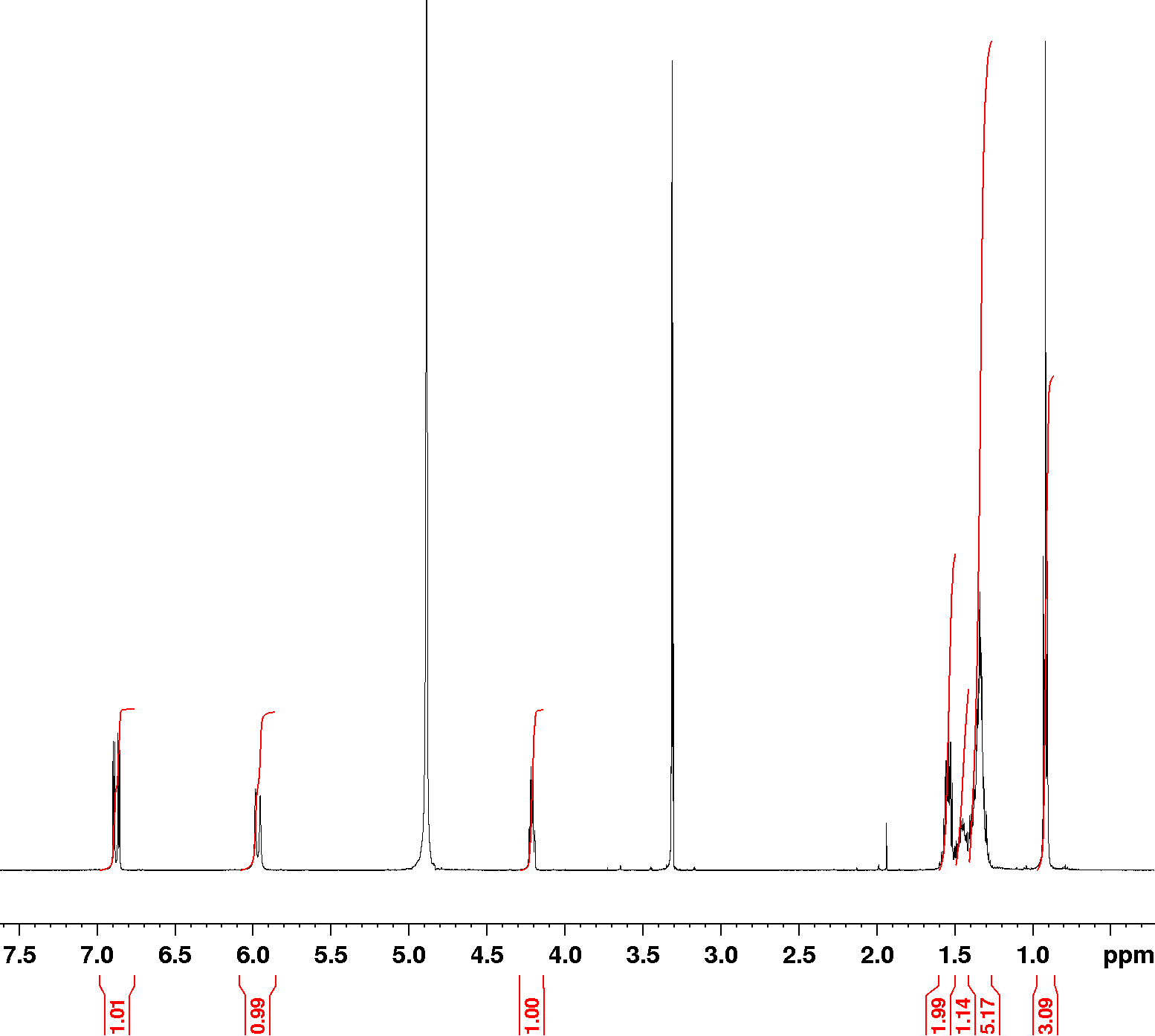 |
| --- |
| Supplementary Figure 35. ^1^H NMR spectrum of compound 5 |

| 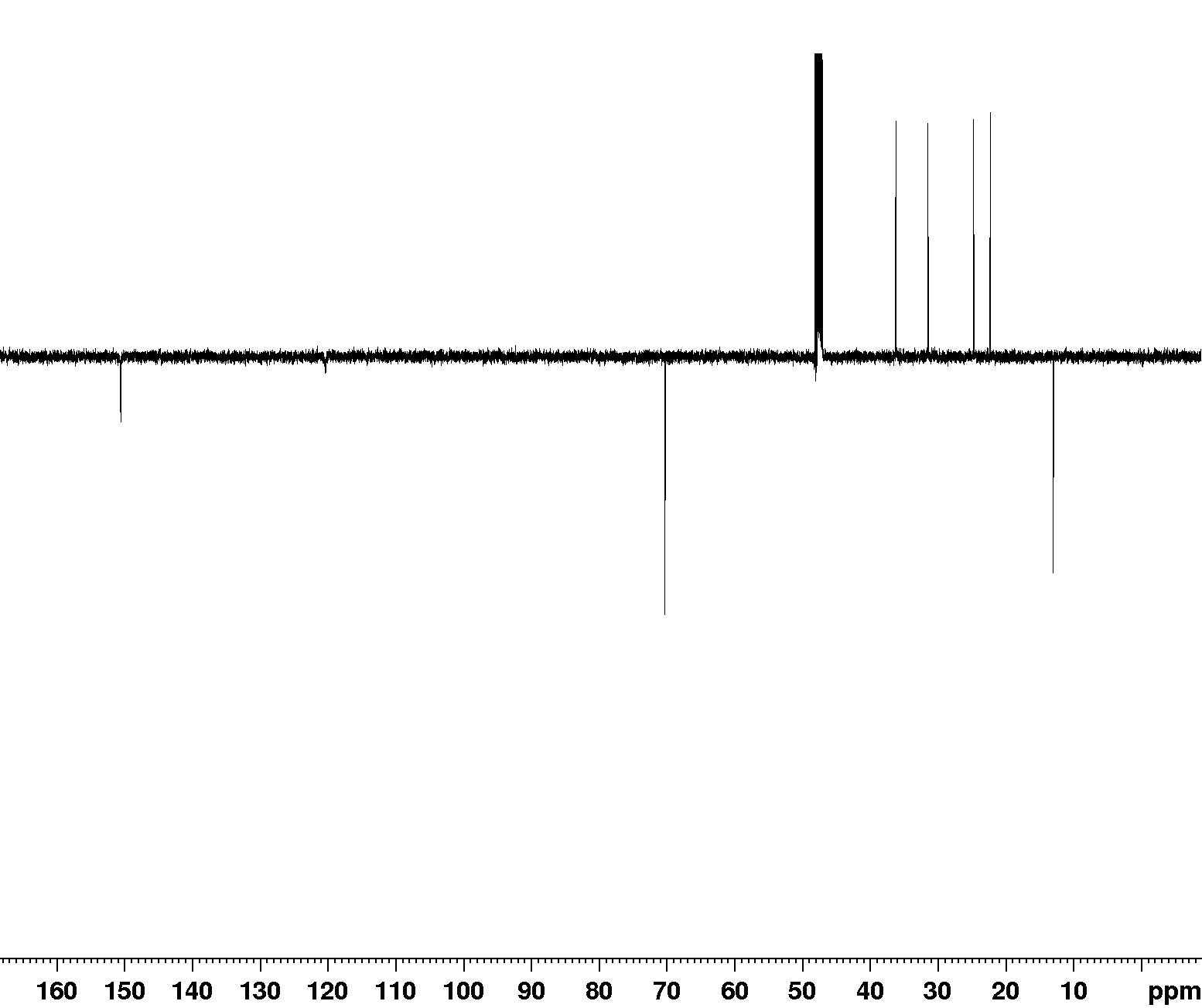 |
| --- |
| Supplementary Figure 36. ^13^C NMR spectrum (DEPTq) of compound 5 |

| 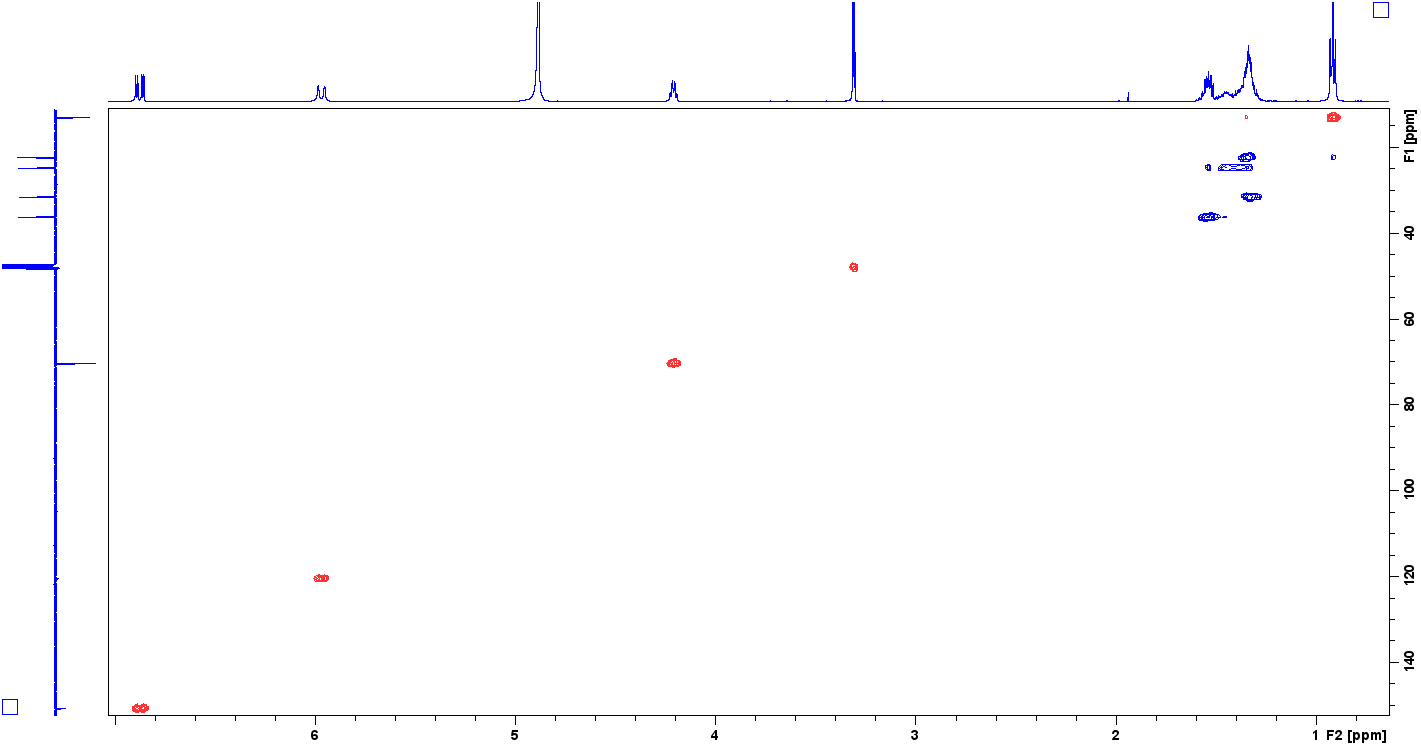 |
| --- |
| Supplementary Figure 37. DEPT-edited HSQC spectrum of compound 5 |

| 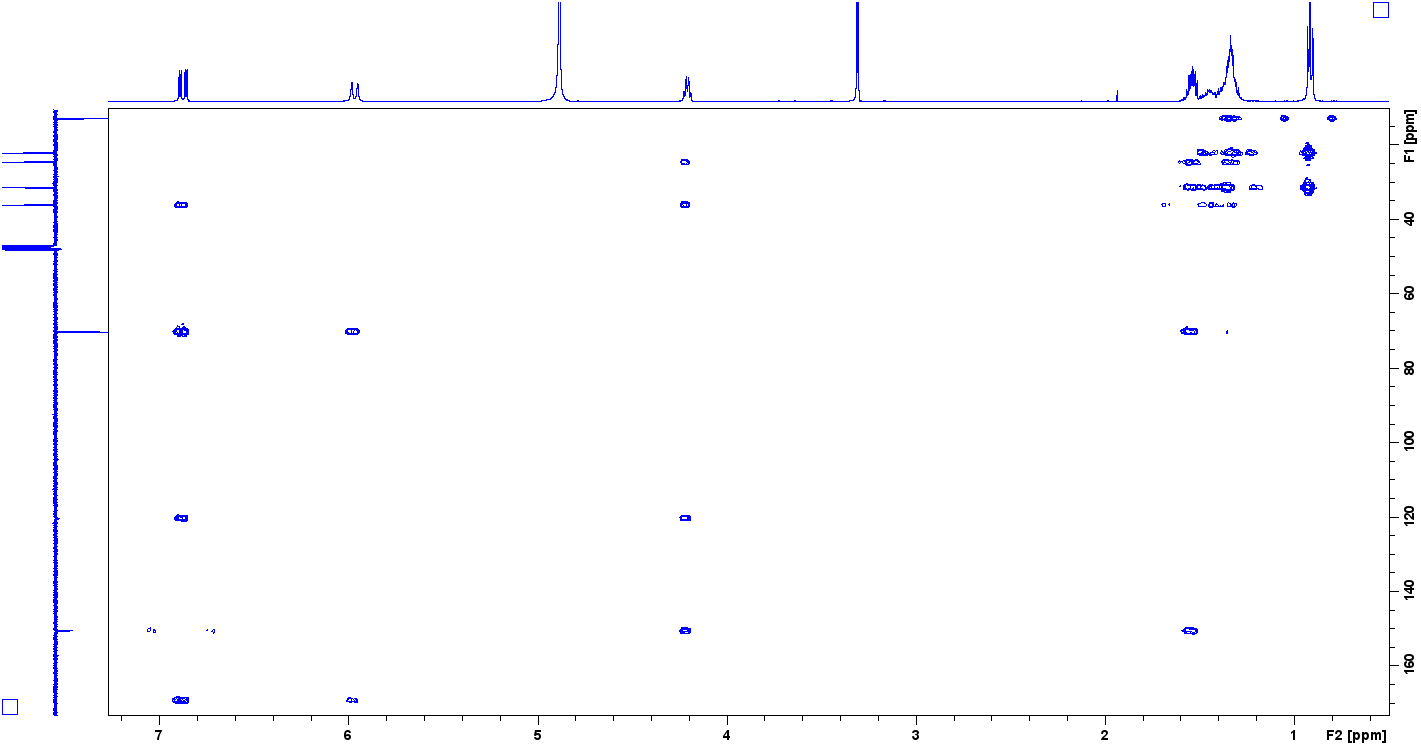 |
| --- |
| Supplementary Figure 38. HMBC spectrum of compound 5 |

# Spectra and spectral data on betulin (Compound 6)

HRESIMS *m/z* 443.3868 [M + H]+ (Δ 3.5 ppm; C_30_H_51_O_2_); HRESI-MSMS (CID = 15%, 30%, 45%) *m/z* 425.3764, 407.3660, 395.3663, 351.3053, 283.2407, 257.2257, 227.1787, 217.1944.

|  |
| --- |
| Supplementary Figure 39. HRESI-MS spectrum of compound 6 in positive ionization mode |

|  |
| --- |
| Supplementary Figure 40. MS-MS spectrum of compound 6 in positive ionization mode |
| 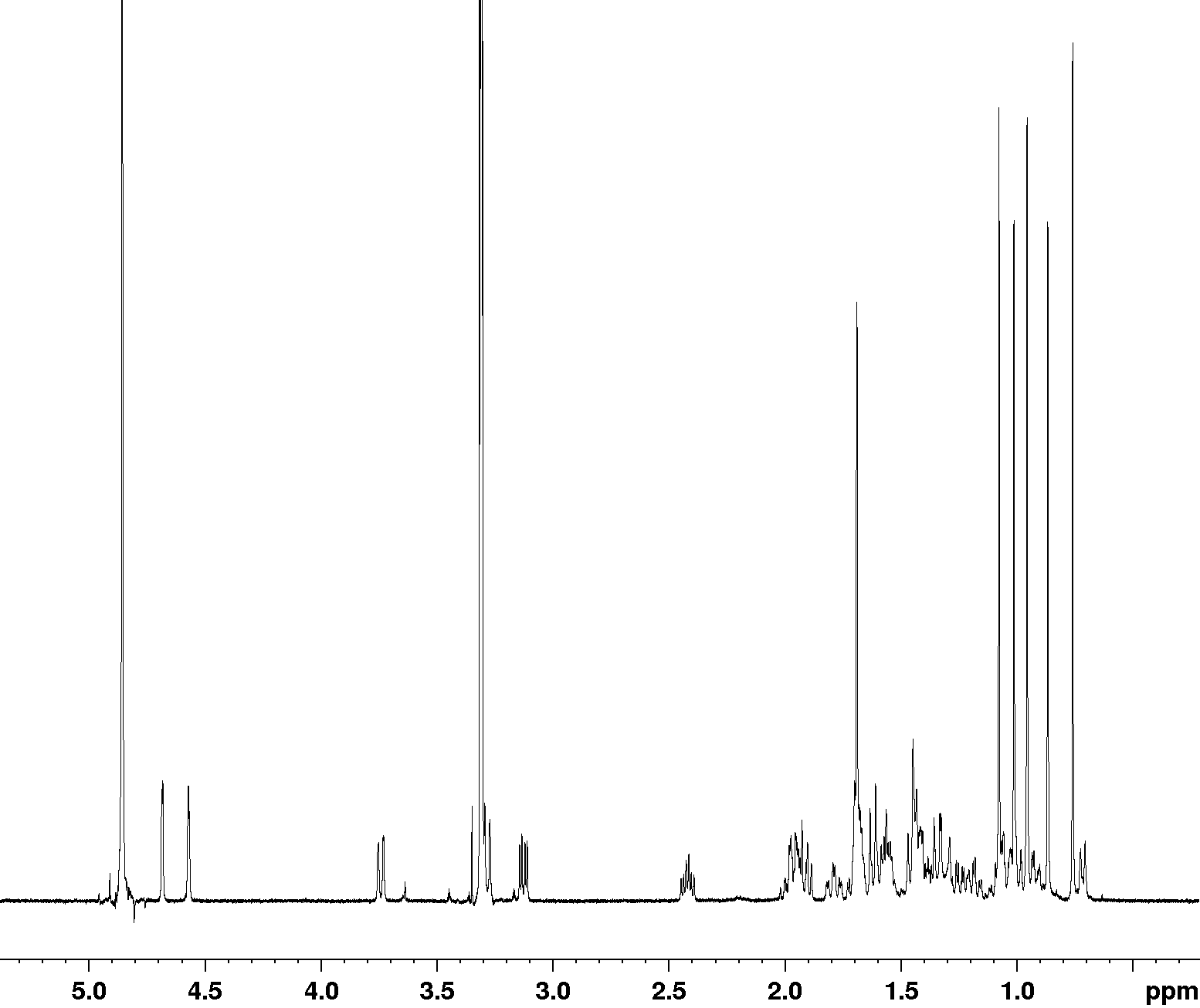 |
| Supplementary Figure 41. ^1^H NMR spectrum of compound 6 |

| 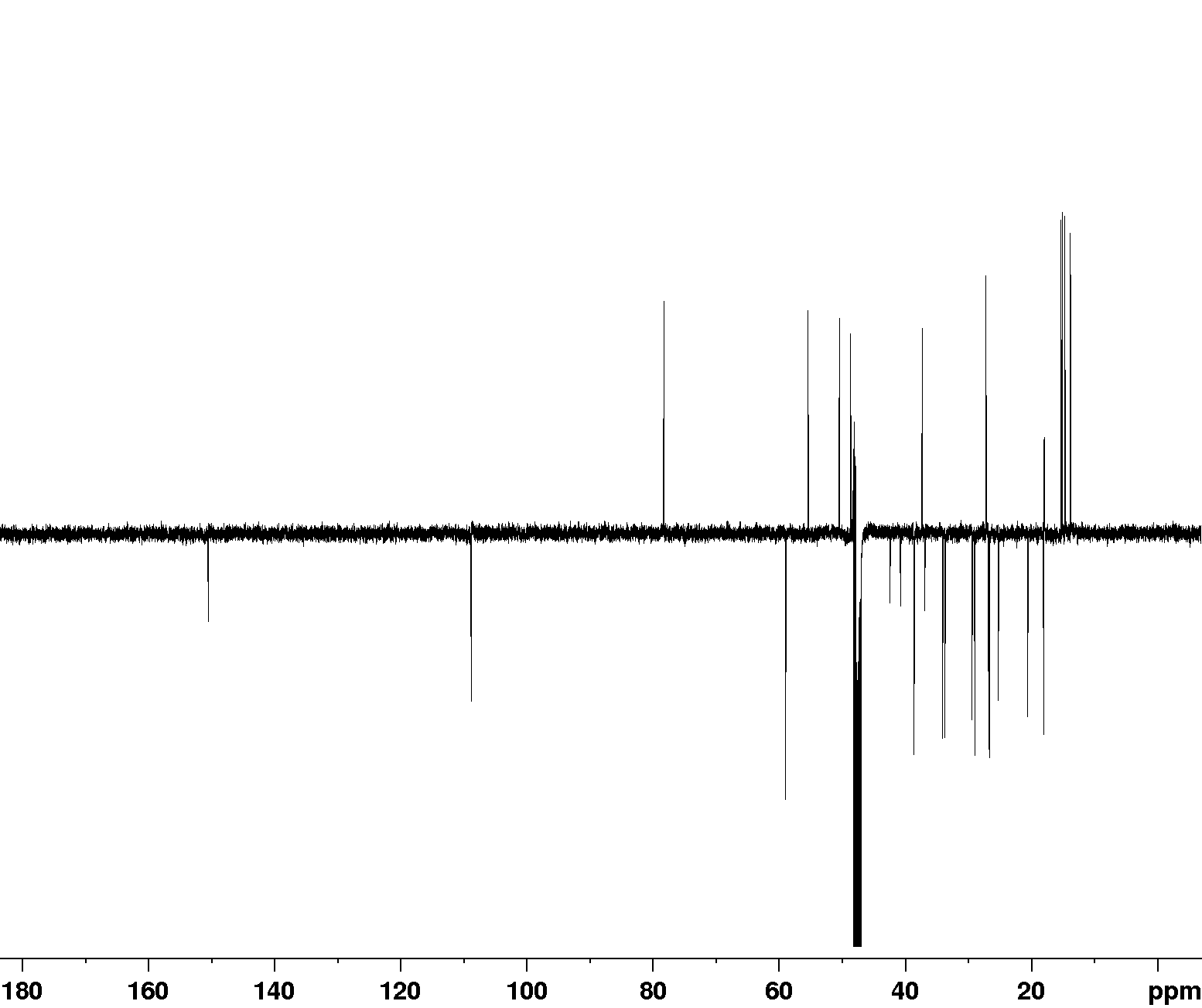 |
| --- |
| Supplementary Figure 42. ^13^C NMR spectrum (DEPTq) of compound 6 |

| 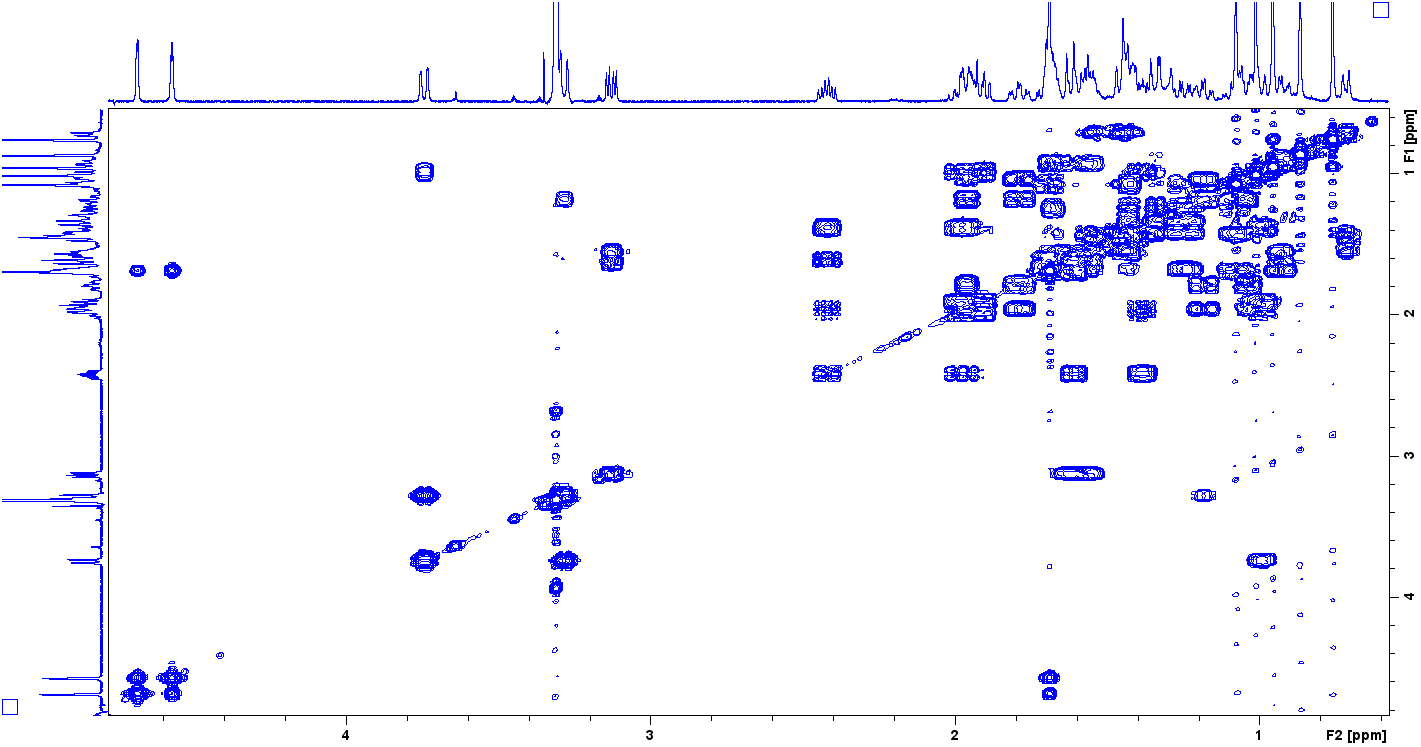 |
| --- |
| Supplementary Figure 43. COSY spectrum of compound 6 |

| 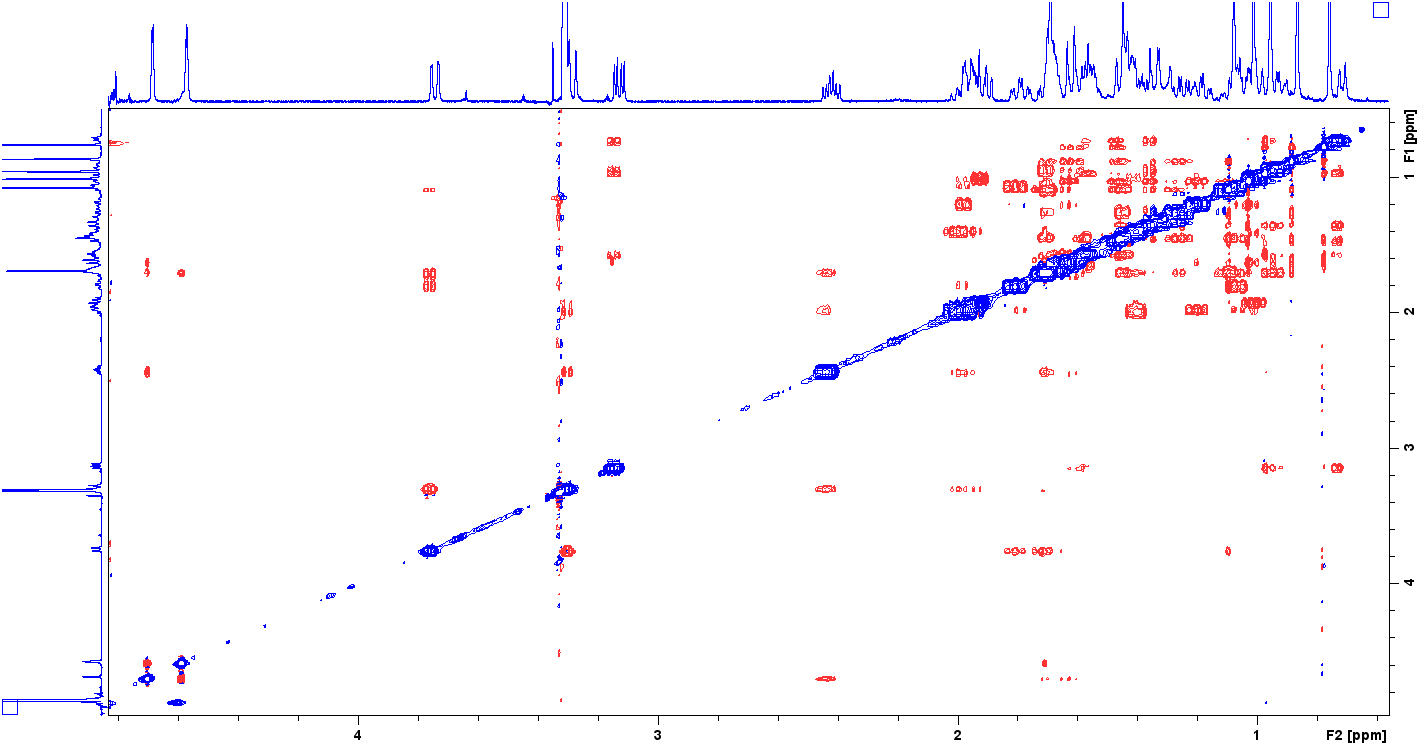 |
| --- |
| Supplementary Figure 44. NOESY spectrum of compound 6 |

| 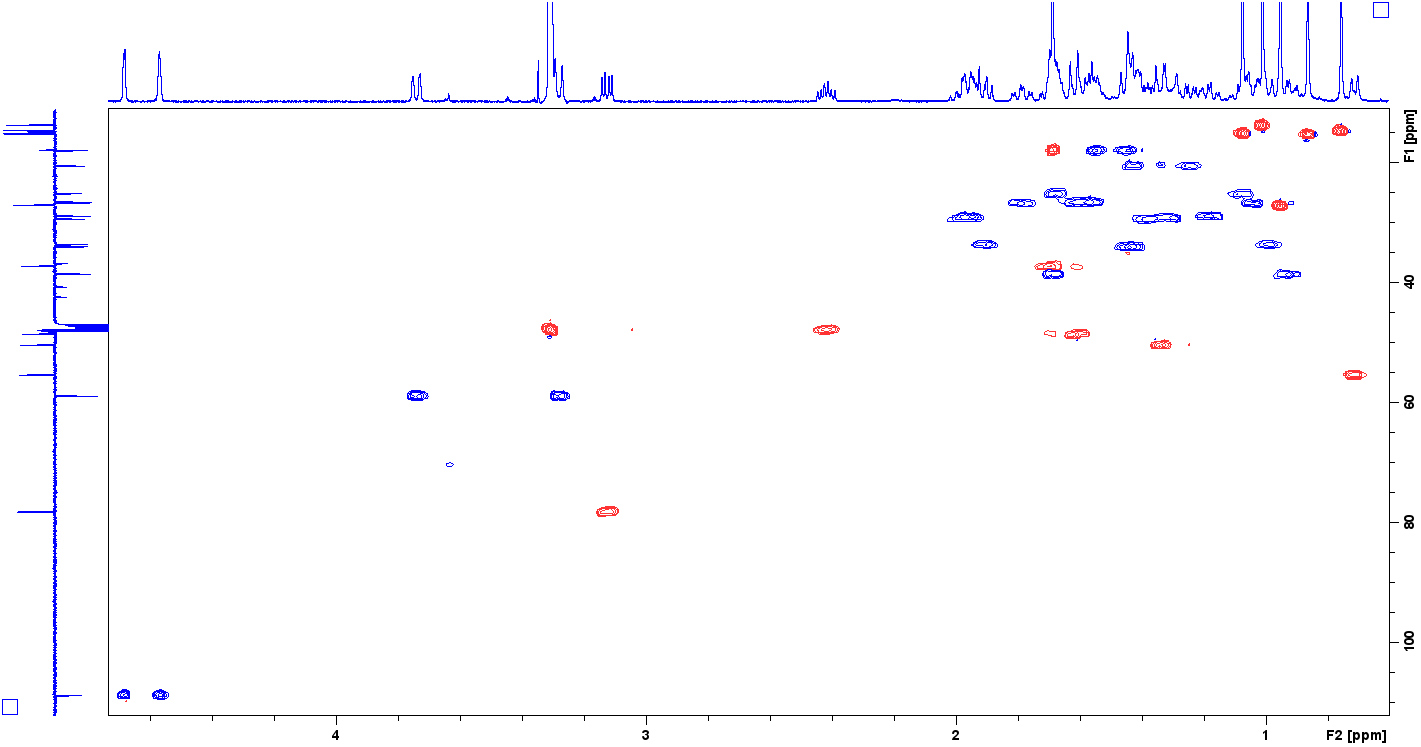 |
| --- |
| Supplementary Figure 45. DEPT-edited HSQC spectrum of compound 6 |

| 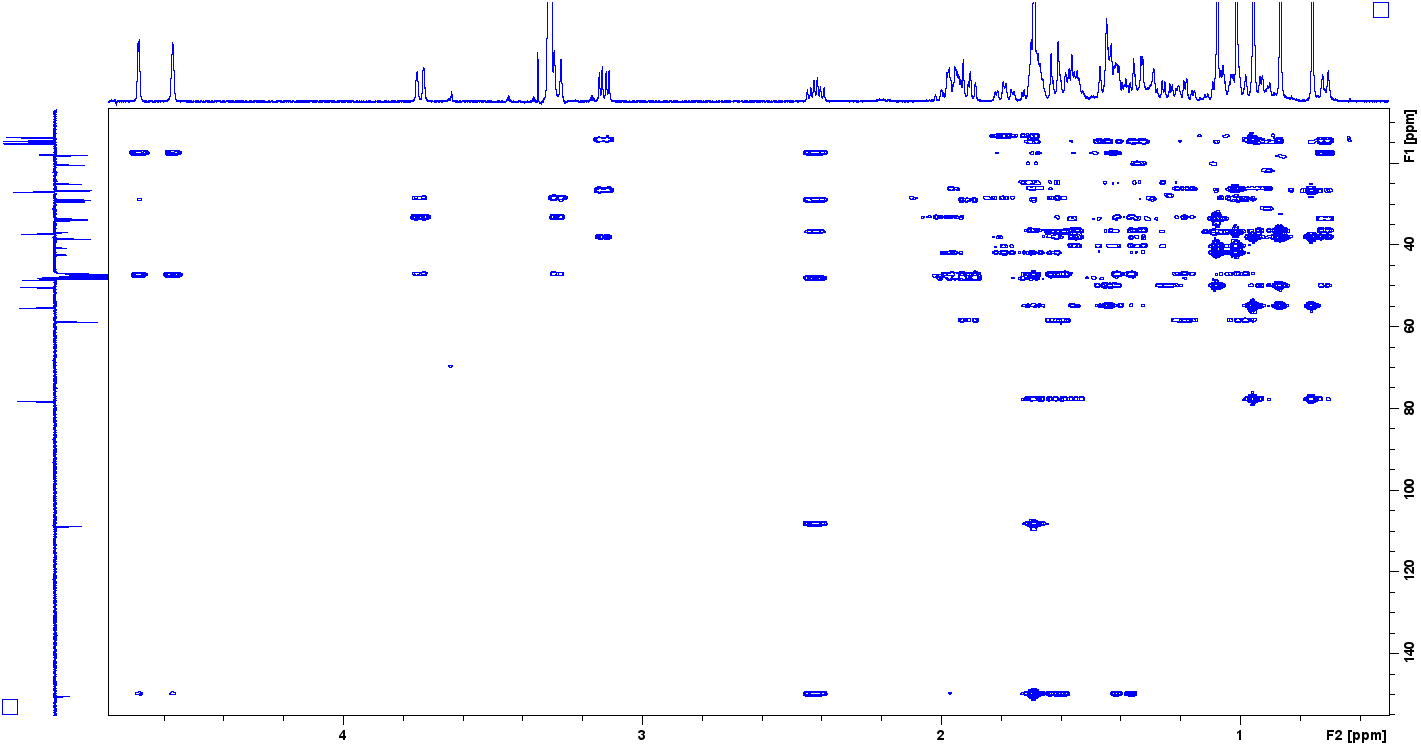 |
| --- |
| Supplementary Figure 46. HMBC spectrum of compound 6 |

# Spectra and spectral data on ergosterol peroxide (Compound 7)

HRESIMS *m/z* 429.3359 [M + H]+ (Δ 1.0 ppm; C_28_H_45_O_3_); HRESI-MSMS (CID = 15%, 30%, 45%) *m/z* 395.3301, 377.3196

|  |
| --- |
| Supplementary Figure 47. HRESI-MS spectrum of compound 7 in positive ionization mode |

|  |
| --- |
| Supplementary Figure 48. MS-MS spectrum of compound 7 in positive ionization mode |
| 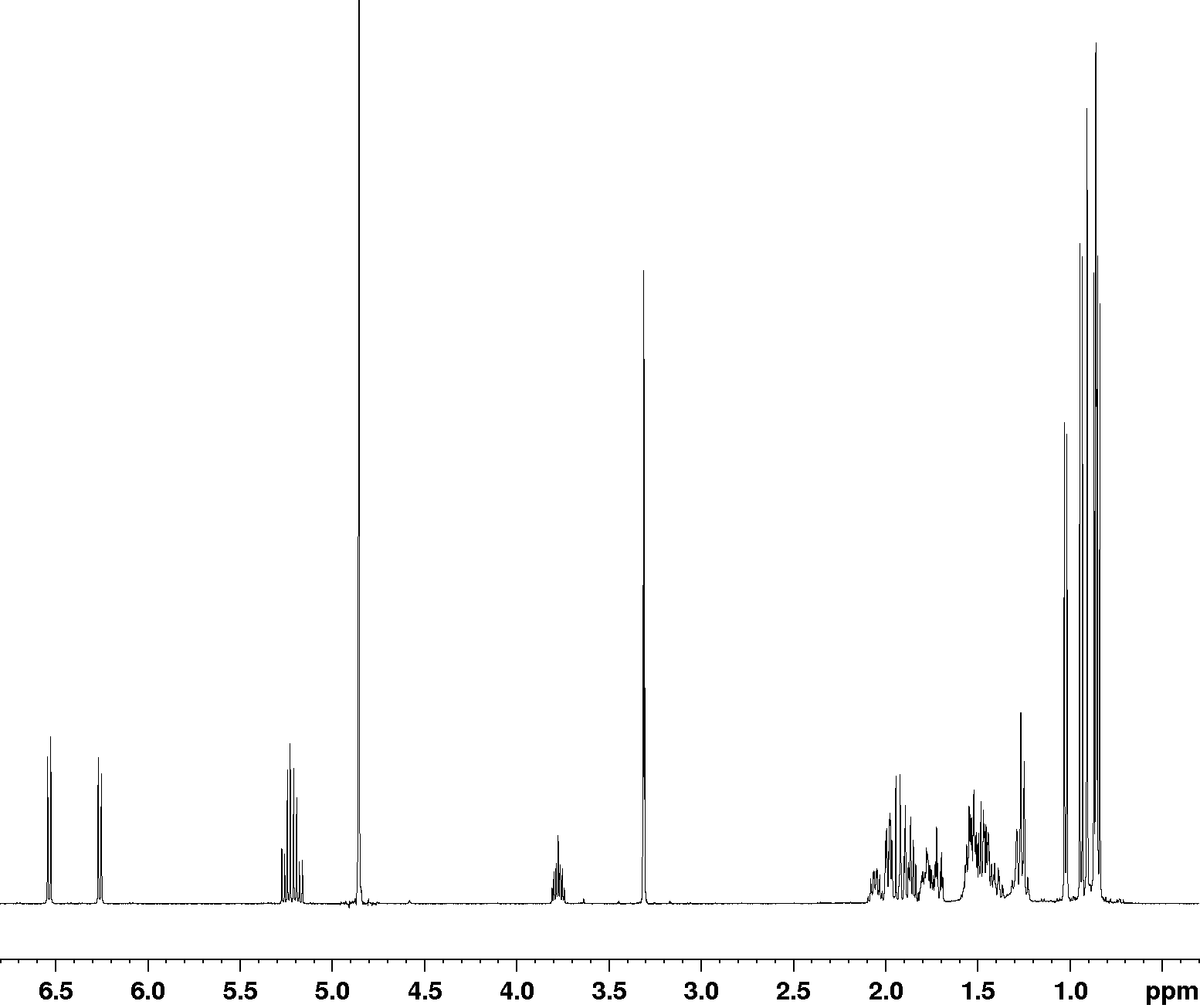 |
| Supplementary Figure 49. ^1^H NMR spectrum of compound 7 |

| 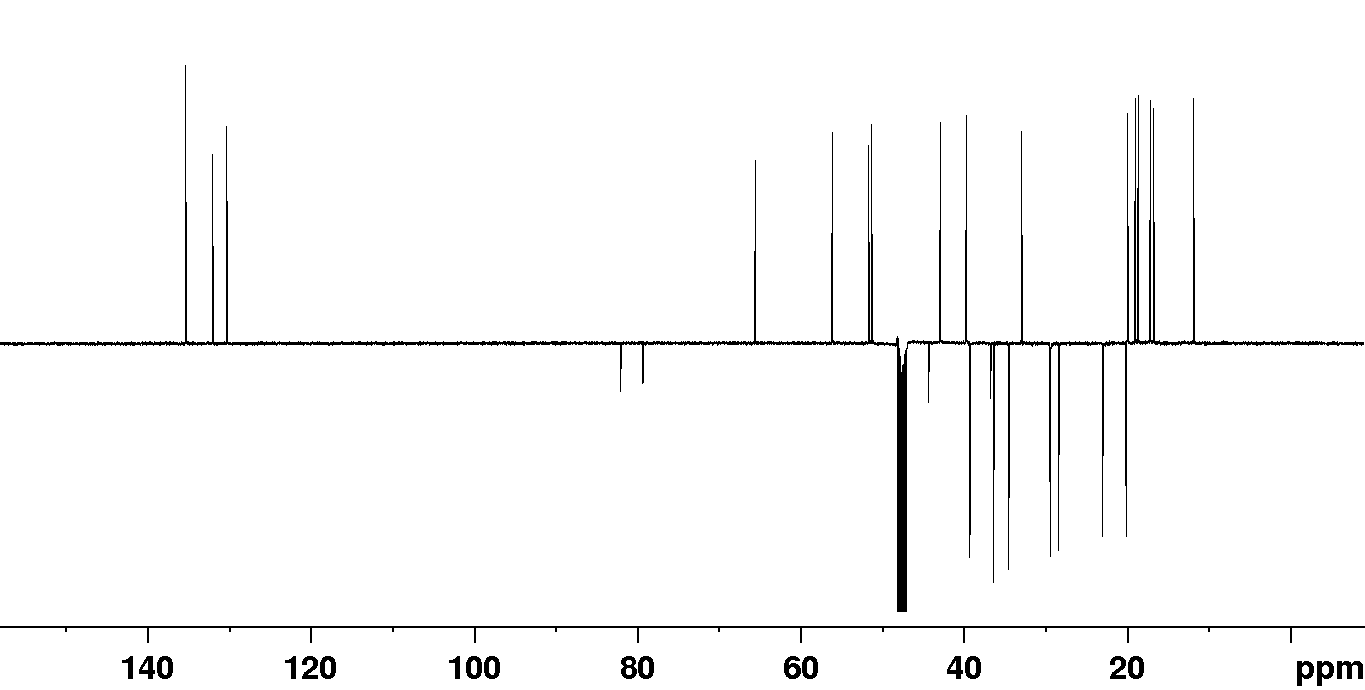 |
| --- |
| Supplementary Figure 50. ^13^C NMR spectrum (DEPTq) of compound 7 |

| 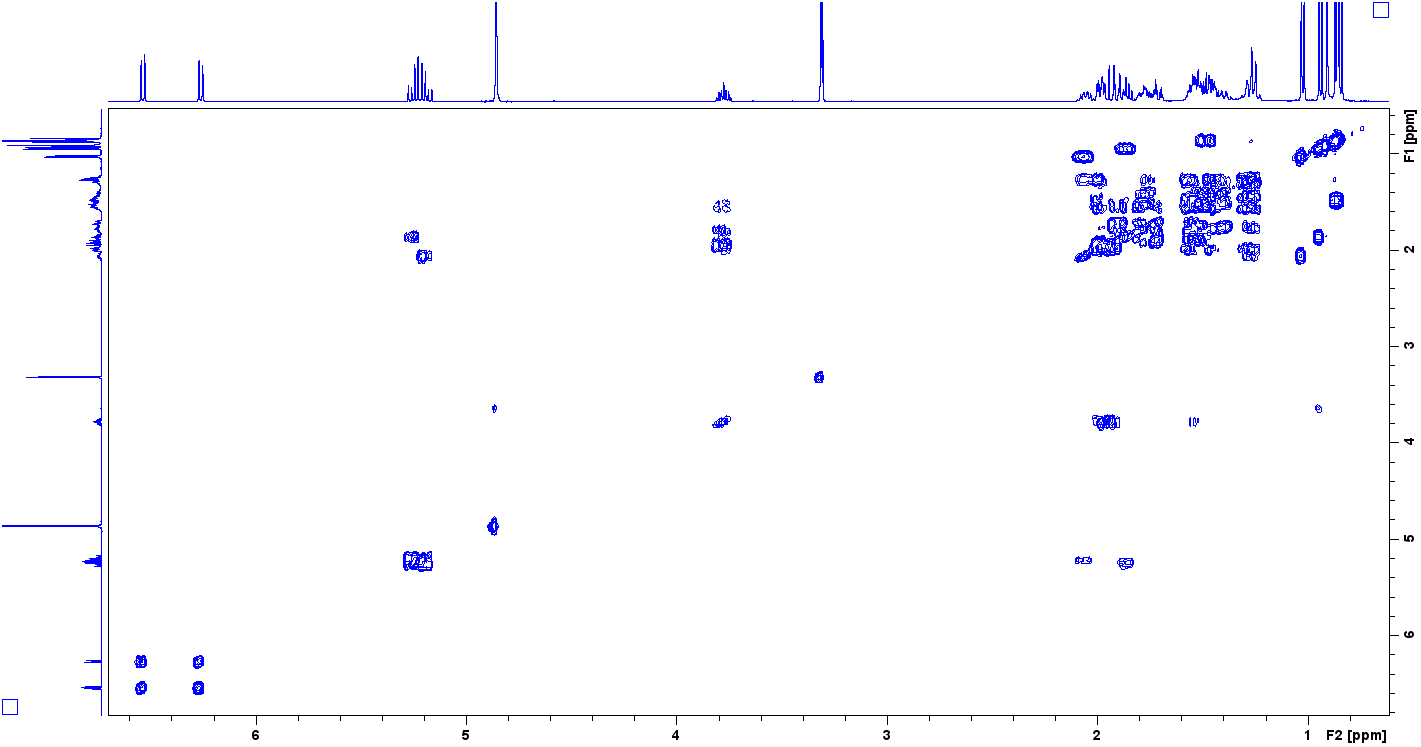 |
| --- |
| Supplementary Figure 51. COSY spectrum of compound 7 |

| 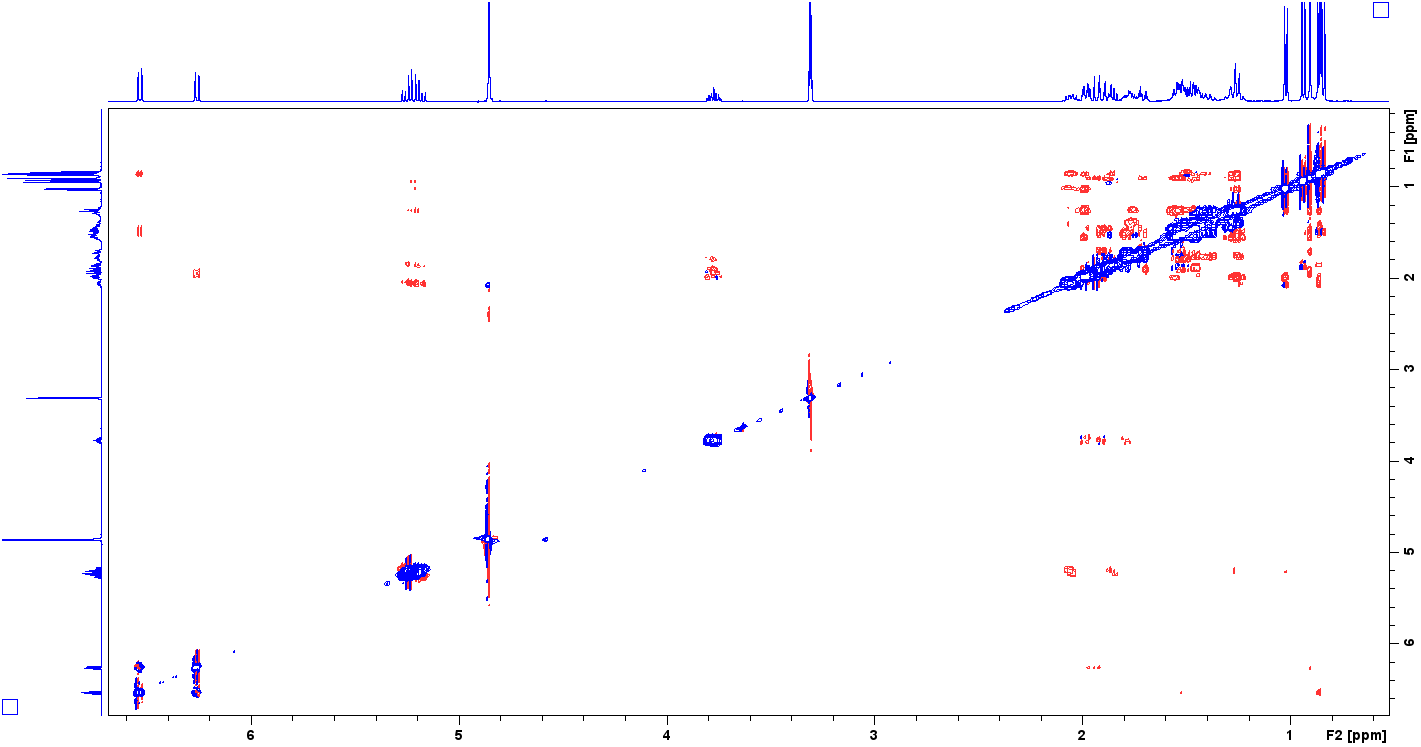 |
| --- |
| Supplementary Figure 52. 2D ROESY spectrum of compound 7 |

| 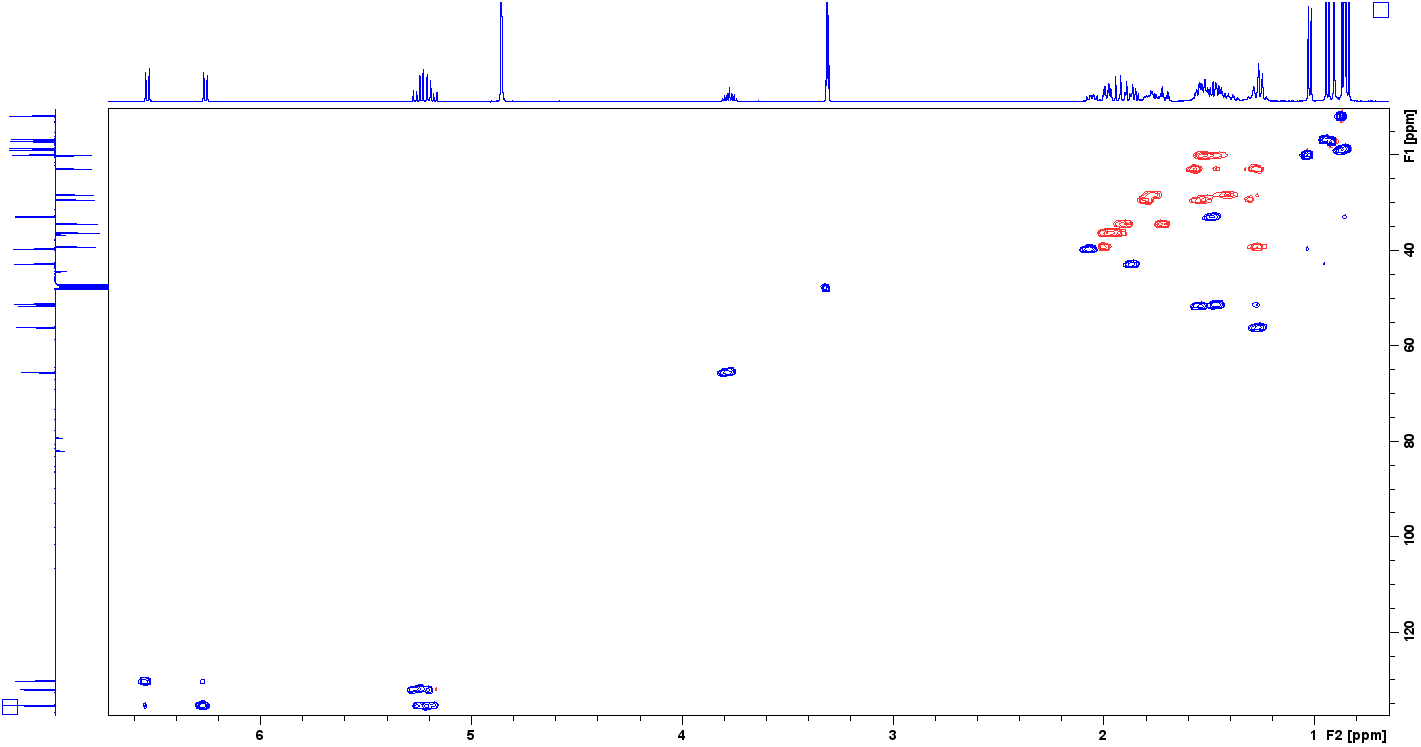 |
| --- |
| Supplementary Figure 53. DEPT-edited HSQC spectrum of compound 7 |

| 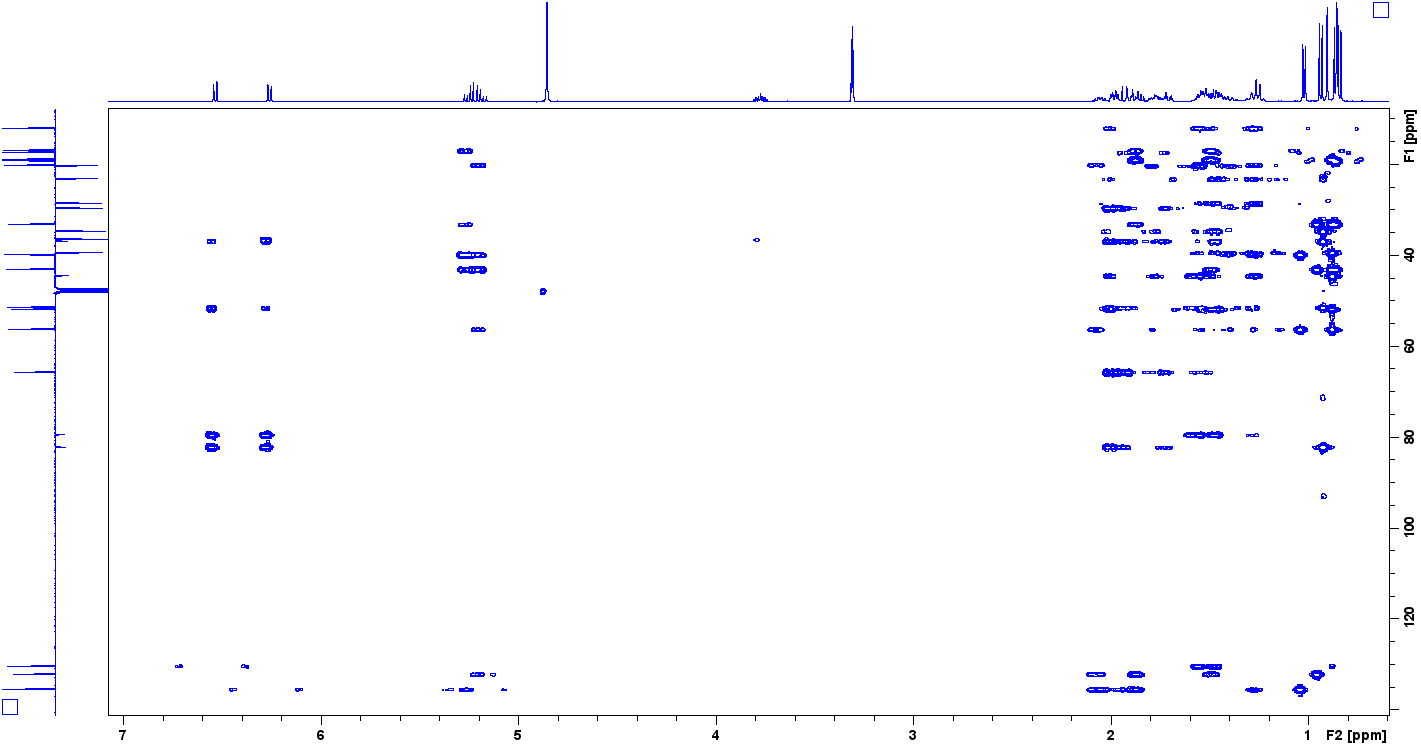 |
| --- |
| Supplementary Figure 54. HMBC spectrum of compound 7 |

# Spectra and spectral data on ergosterol peroxide glucoside (Compound 8)

HRESIMS *m/z* 591.3883 [M + H]+ (Δ 1.4 ppm; C_34_H_55_O_8_); HRESI-MSMS (CID = 15%, 30%, 45%) *m/z* 573.3779, 555.3675, 429.3362, 411.3255, 395.3266, 377.3200.

|  |
| --- |
| Supplementary Figure 55. HRESI-MS spectrum of compound 8 in positive ionization mode |

|  |
| --- |
| Supplementary Figure 56. MS-MS spectrum of compound 8 in positive ionization mode |

| 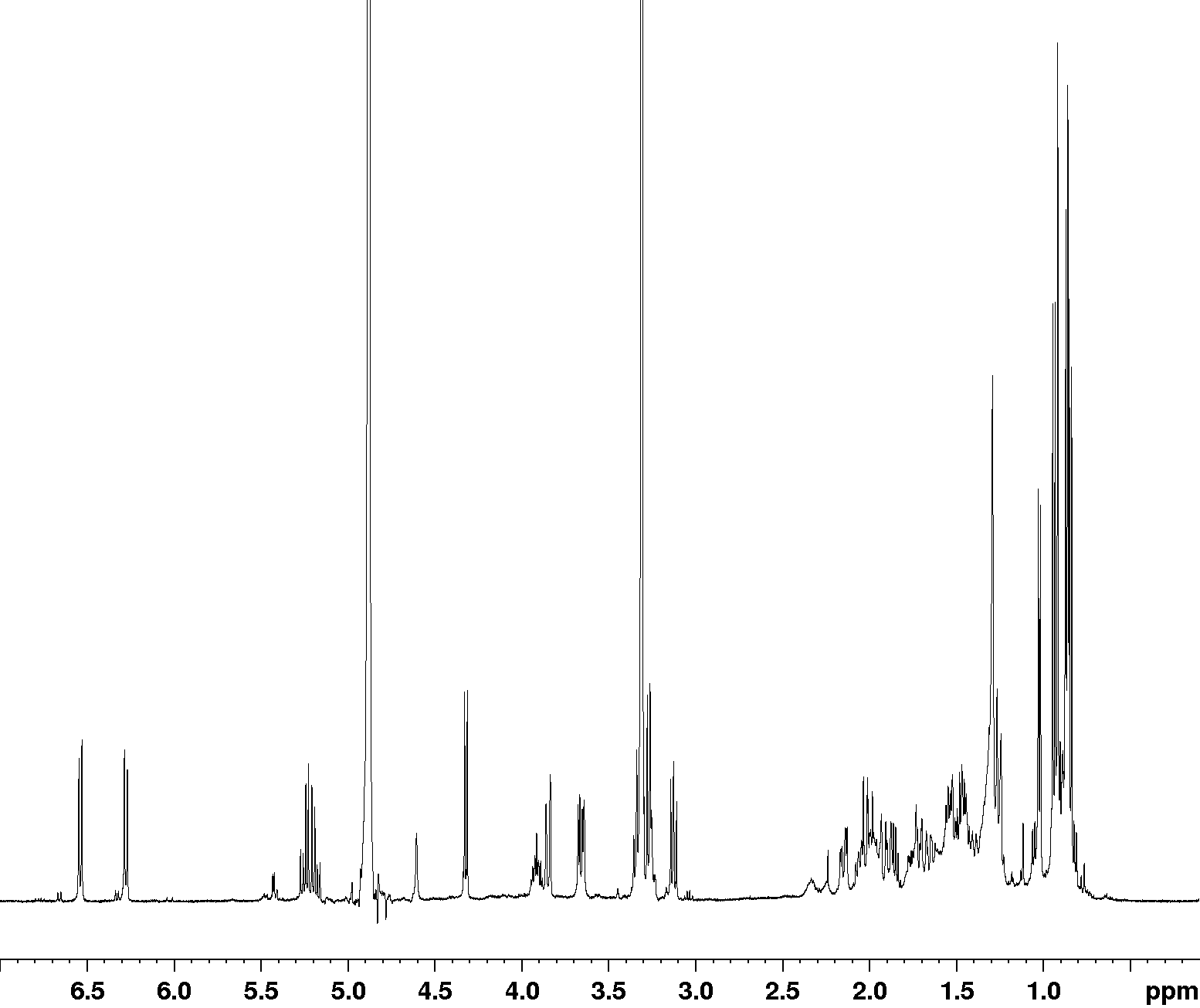 |
| --- |
| Supplementary Figure 57. ^1^H NMR spectrum of compound 8 |

| 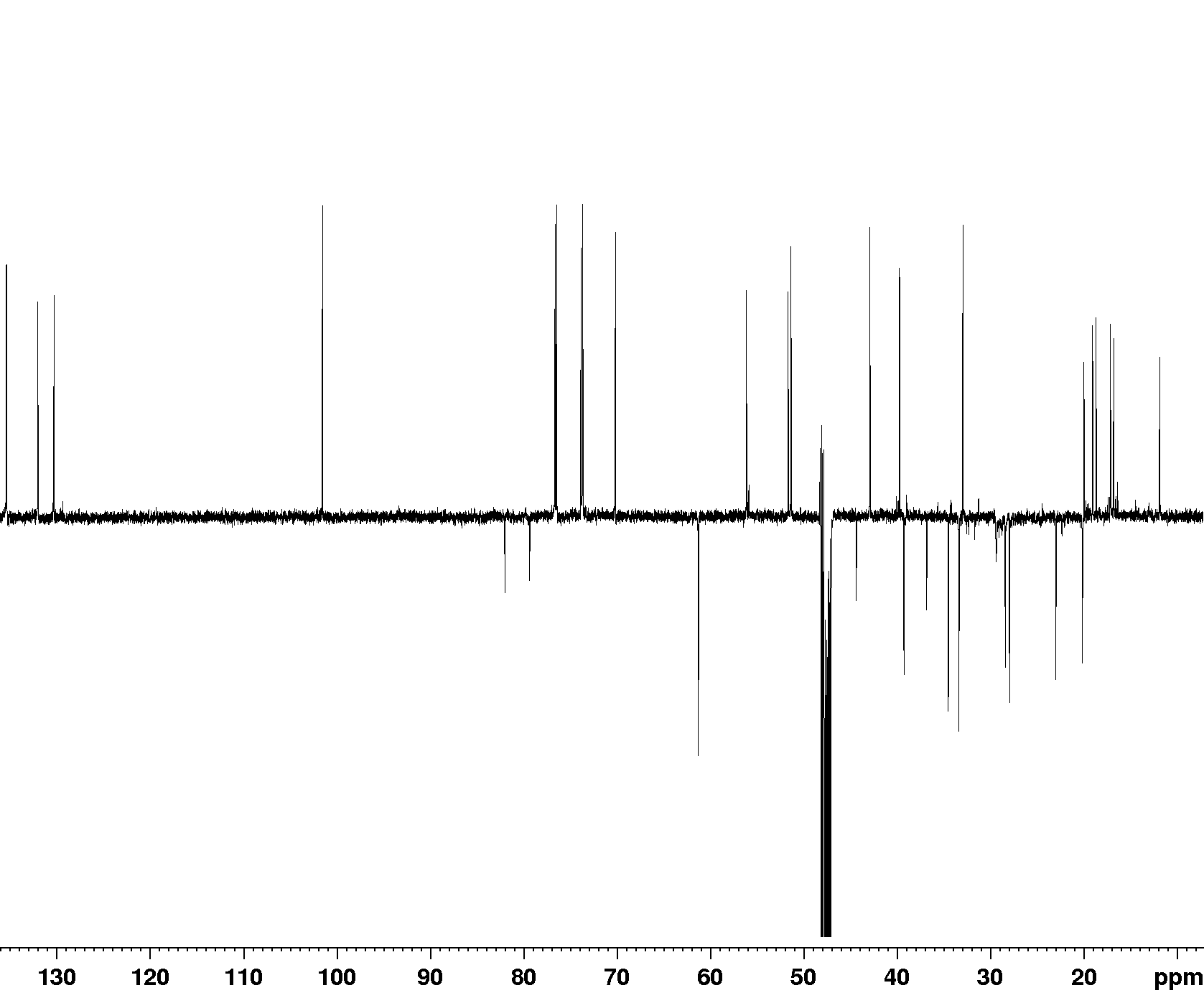 |
| --- |
| Supplementary Figure 58. ^13^C NMR spectrum (DEPTq) of compound 8 |

| 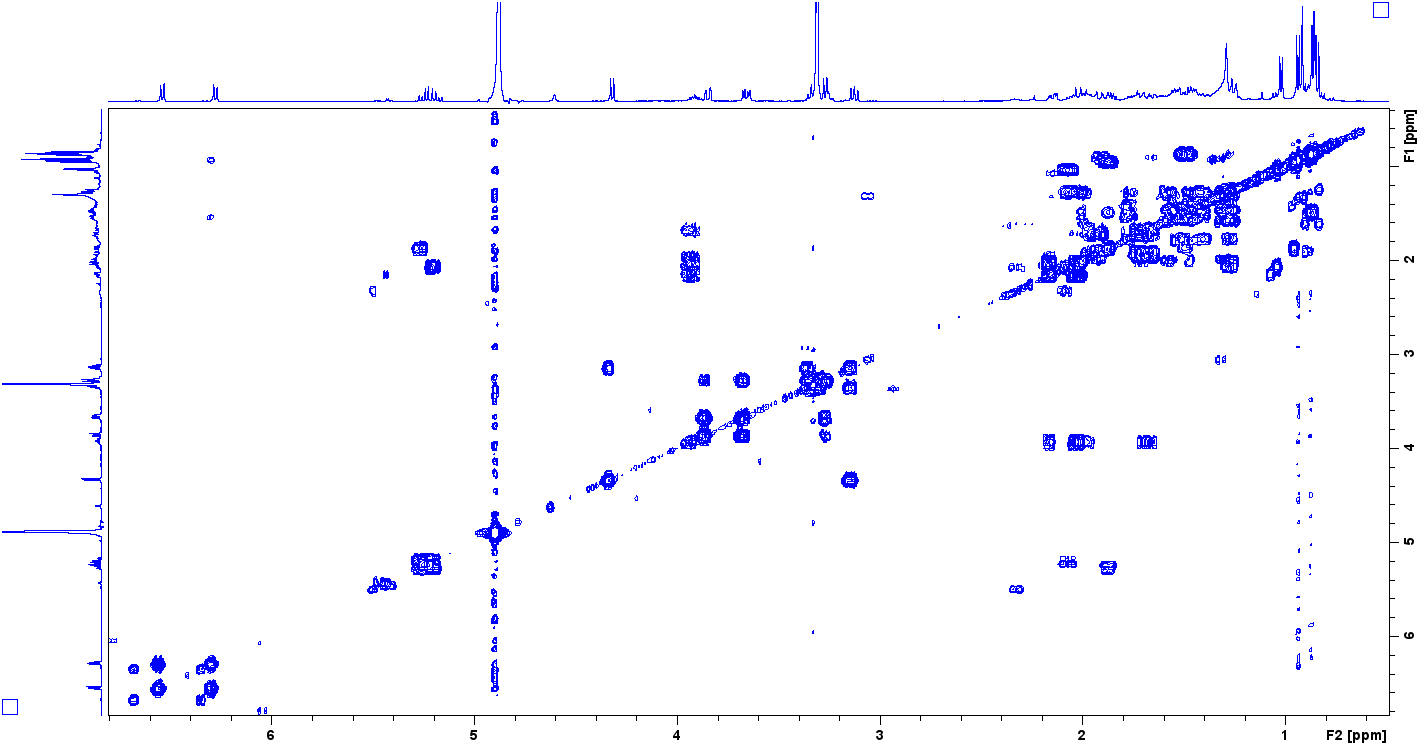 |
| --- |
| Supplementary Figure 59. COSY spectrum of compound 8 |

| 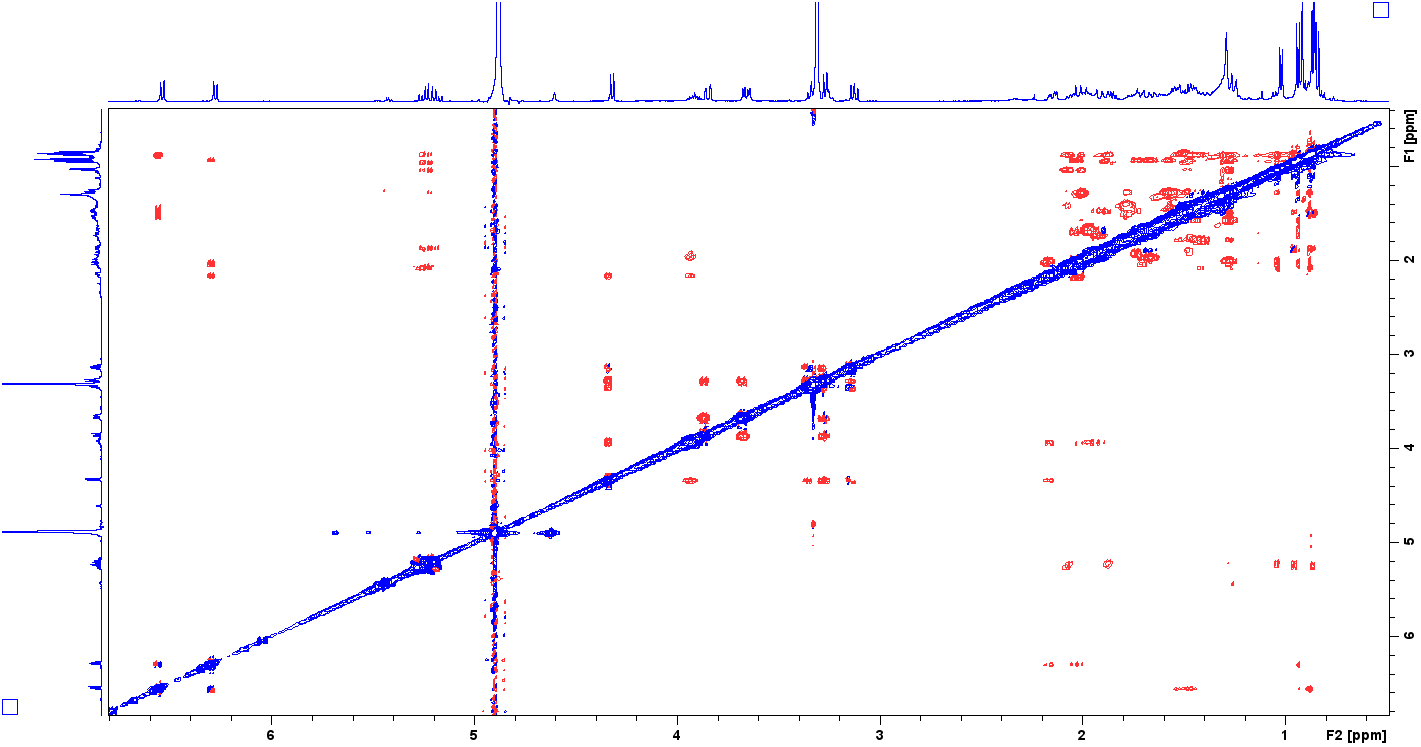 |
| --- |
| Supplementary Figure 60. NOESY spectrum of compound 8 |

| 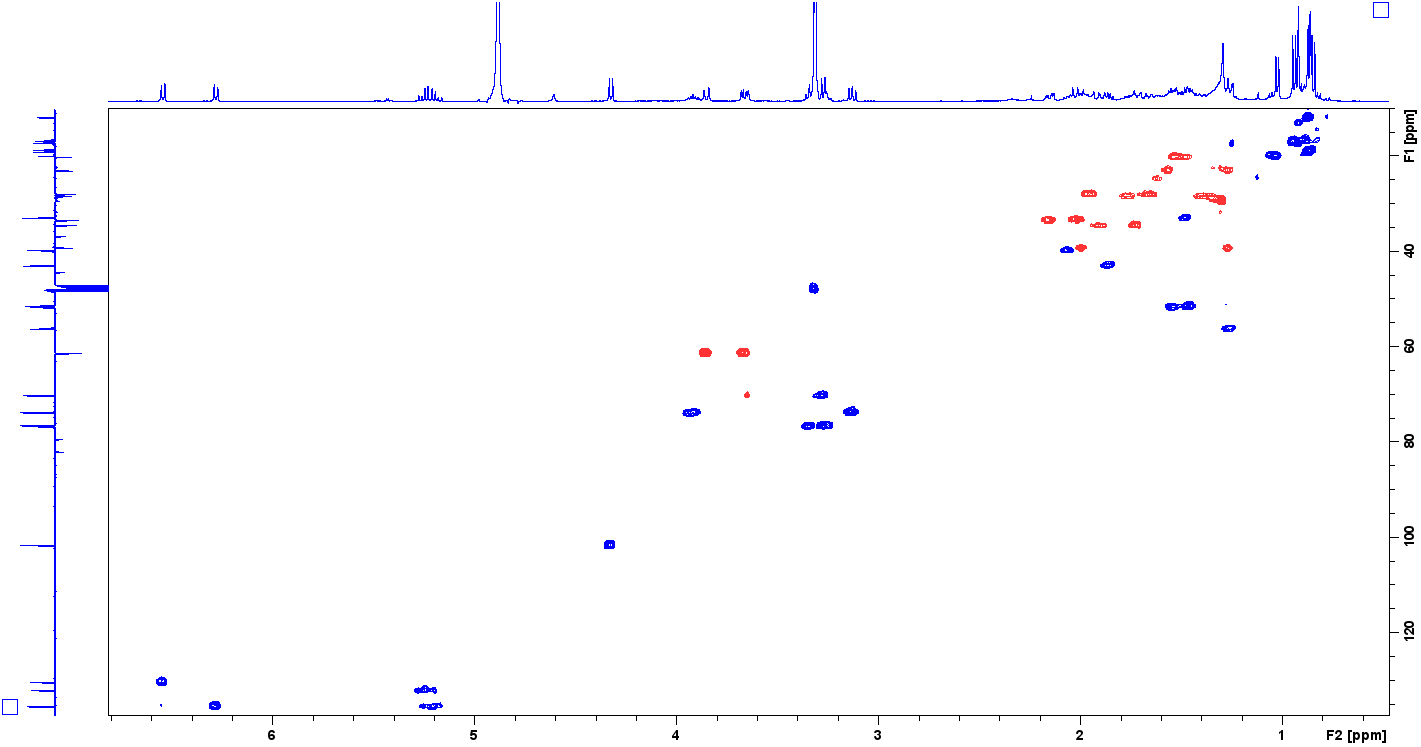 |
| --- |
| Supplementary Figure 61. DEPT-edited HSQC spectrum of compound 8 |

| 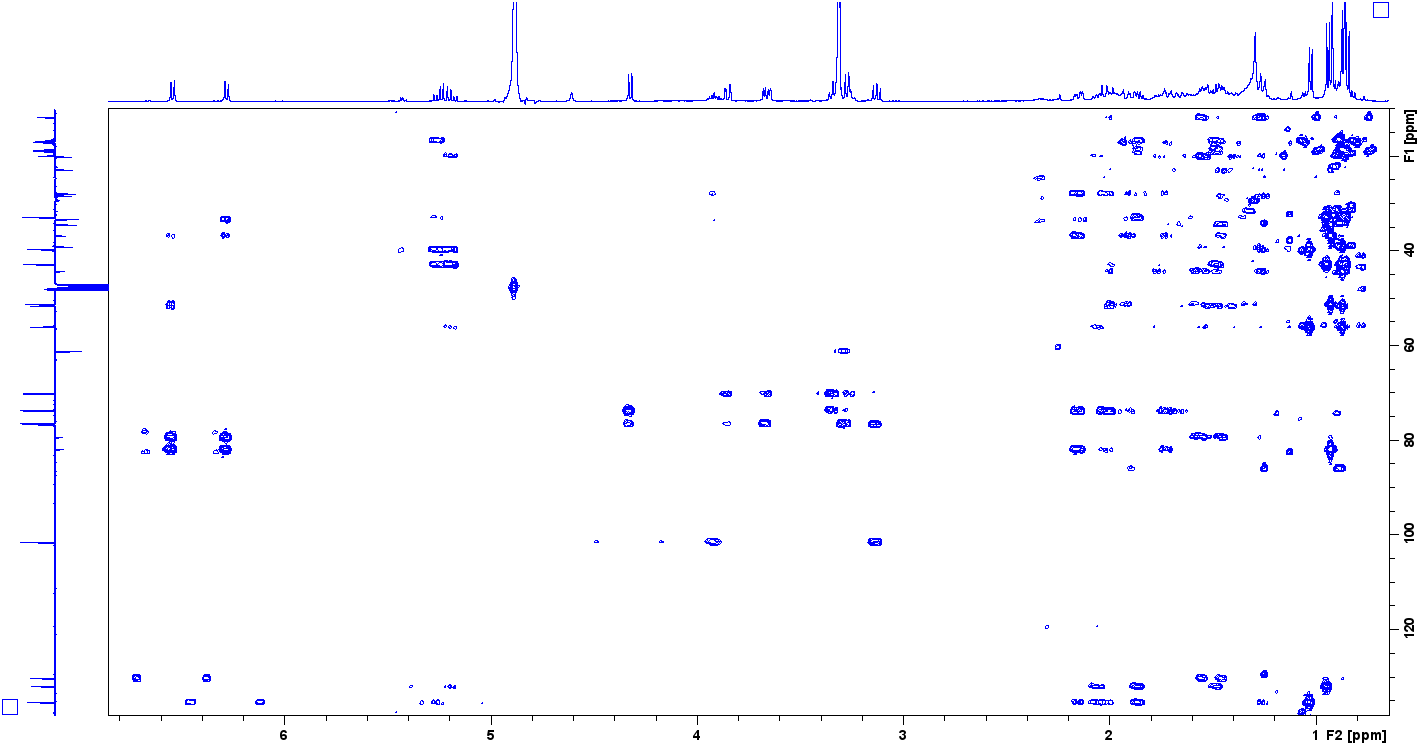 |
| --- |
| Supplementary Figure 62. HMBC spectrum of compound 8 |
